# Nuclear DLC1 exerts oncogenic function through association with FOXK1 for cooperative activation of MMP9 expression in melanoma

**DOI:** 10.1038/s41388-020-1274-8

**Published:** 2020-03-25

**Authors:** Xintao Yang, Feng Hu, Jessica Aijia Liu, Shan Yu, May Pui Lai Cheung, Xuelai Liu, Irene Oi-Lin Ng, Xin-Yuan Guan, Kelvin K. W. Wong, Rakesh Sharma, Hong Lok Lung, Yufei Jiao, Leo Tsz On Lee, Martin Cheung

**Affiliations:** 1 Shenzhen Institute of Research and Innovation (HKU-SIRI), The University of Hong Kong, Shenzhen, China; 2 School of Biomedical Sciences, Li Ka Shing Faculty of Medicine, The University of Hong Kong, Hong Kong, China; 3 Department of Anaesthesiology, Li Ka Shing Faculty of Medicine, The University of Hong Kong, Hong Kong, China; 4 Cancer Centre, Faculty of Health Sciences, University of Macau, Taipa, Macau, China; 5 Department of Pediatric Surgery, Second Hospital of Hebei Medical University, Shijiazhuang, Hebei China; 6 State Key Laboratory of Liver Research and Department of Pathology, Li Ka Shing Faculty of Medicine, The University of Hong Kong, Hong Kong, China; 7 Department of Clinical Oncology, Li Ka Shing Faculty of Medicine, The University of Hong Kong, Hong Kong, China; 8 Centre for PanorOmic Sciences, Proteomics and Metabolomics Core Facility, Li Ka Shing Faculty of Medicine, The University of Hong Kong, Hong Kong, China; 9 Department of Biology, Faculty of Science, Hong Kong Baptist University, Hong Kong, China; 10 Department of Pathology, The Second Affiliated Hospital of Harbin Medical University, Harbin, China; 11 Centre of Reproduction, Development and Aging, Faculty of Health Sciences, University of Macau, Taipa, Macau, China

**Keywords:** Melanoma, Oncogenes

## Abstract

A Rho GTPase-activating protein (RhoGAP), deleted in liver cancer 1 (DLC1), is known to function as a tumor suppressor in various cancer types; however, whether *DLC1* is a tumor-suppressor gene or an oncogene in melanoma remains to be clarified. Here we revealed that high DLC1 expression was detected in most of the melanoma tissues where it was localized in both the nuclei and the cytoplasm. Functional studies unveiled that DLC1 was both required and sufficient for melanoma growth and metastasis. These tumorigenic events were mediated by nuclear-localized DLC1 in a RhoGAP-independent manner. Mechanistically, mass spectrometry analysis identified a DLC1-associated protein, FOXK1 transcription factor, which mediated oncogenic events in melanoma by translocating and retaining DLC1 into the nucleus. RNA-sequencing profiling studies further revealed MMP9 as a direct target of FOXK1 through DLC1-regulated promoter occupancy for cooperative activation of MMP9 expression to promote melanoma invasion and metastasis. Concerted action of DLC1–FOXK1 in MMP9 gene regulation was further supported by their highly correlated expression in melanoma patients’ samples and cell lines. Together, our results not only unravel a mechanism by which nuclear DLC1 functions as an oncogene in melanoma but also suggest an unexpected role of RhoGAP protein in transcriptional regulation.

## Introduction

Melanoma is considered the most aggressive form of skin cancer, accounting for over 80% of skin cancer deaths in the United States [1]. The high mortality rate among melanoma patients is directly related to the metastatic properties of the tumor. Over 50% of melanoma patients harbor the BRAF mutation, which leads to constitutive activation of the oncogenic signaling pathways together with dysregulated expression of cancer driver genes, resulting in the neoplastic transformation of embryonic neural crest (NC)-derived melanocytes located in the basal layer of the skin epidermis into metastatic melanomas [2, 3]. Despite improved overall survival of patients with metastatic melanoma treated with an inhibitor to target BRAF mutation, cancer can rapidly acquire resistance to the treatment that limits its long-term efficacy [4]. Therefore, there is an urgent need to identify new factors in governing melanoma colonization as alternative therapeutic targets.

The NC is a transient and multipotent stem cell-like population that acquire migratory capacity upon delamination from the dorsal neural tube followed by colonizing to various embryonic regions where they give rise to the peripheral nervous system, craniofacial skeleton, and melanocytes [5]. Emerging evidence suggests that melanoma cells hijack the NC developmental program for their growth and metastasis [3, 6]. For instance, transcription factor SOX10 is a key regulator for the specification and homeostasis of embryonic and adult melanocytes, respectively [7, 8]. Consistently, SOX10 is functionally required for the initiation, proliferation, and invasion of melanoma [9–12]. In contrast, a closely related gene *SOX9* is not involved in any stages of melanocyte development, though it has an early role in NC specification and migration [13, 14]. Our recent studies revealed that a low level of SOX9 expression inhibited melanoma growth and metastasis, whereas high SOX9 promoted melanoma progression [15]. Besides, we also demonstrated that the asymmetric location of deleted in liver cancer 1 (DLC1) is essential for the migration of trunk NC cells in a directional manner [16]. However, a previous report showed that loss of DLC1 expression in primary cutaneous and metastatic melanomas was associated with poor survival [17], suggesting that DLC1 might inhibit melanoma growth and progression. Whether DLC1 indeed functions as a tumor suppressor in melanoma remains to be determined.

The Rho GTPase-activating protein (RhoGAP), *DLC1*, has been well characterized as a tumor suppressor, as its expression was frequently found to be downregulated in various cancer types, and restoration of its expression in cancer cell lines inhibited tumorigenic growth [18, 19]. The tumor-suppressive function of DLC1 can largely be attributed to its four functional domains: a sterile alpha motif (SAM) at the NH_2_ terminal, a focal adhesion targeting region, a RhoGAP domain, and a steroidogenic acute regulatory protein related lipid-transfer (START) at the COOH terminal. The RhoGAP domain of DLC1 negatively regulates the activity of RhoA, RhoB, RhoC, and to a lesser extent Cdc42 by promoting the hydrolysis of active GTP-bound state into the inactive GDP-bound state [20]. Most of the studies focused on the inhibitory effects of DLC1 on RHOA activity, which is involved in cell proliferation and actin cytoskeletal reorganization for cell migration [21, 22]. A previous report demonstrated that DLC1 inhibited TGF-β-induced expression of parathyroid hormone-like hormone through suppression of Rho-ROCK signaling to prevent breast cancer bone metastasis [23]. In contrast, studies in other cellular contexts showed that interaction of the SAM or the START domain with partner factors contributed to the antitumorigenic functions of DLC1 via a RhoGAP-independent mechanism [24], implying a context-dependent role of DLC1. Apart from its cytoplasmic role in regulating Rho signaling, DLC1 can be localized in the nucleus where it was shown to be less efficient in exerting tumor-suppressor activity in hepatocellular carcinoma [25]. Similarly, DLC1 was also found to be localized in both the cytoplasm and nuclei of metastatic melanomas [17] but how this subcellular localization of DLC1 regulates melanoma progression is largely unknown.

Here, we found elevated levels of *DLC1* mRNA in the majority of melanoma tissues where DLC1 protein was localized in both the nuclei and the cytoplasm. Functional assays showed that nuclear, but not cytoplasmic, localization of DLC1 promoted growth and invasion of melanoma cells in a RhoGAP-independent manner. By proteomic analysis, we identified the Forkhead box transcription factor, Forkhead Box K1 (FOXK1), as a DLC1-associated protein. We demonstrated that FOXK1 was crucial for DLC1 nuclear translocation and retention to orchestrate oncogenic programs in melanoma. Transcriptional profiling using RNA-sequencing identified matrix metalloproteinase 9 (MMP9), which was commonly downregulated in both *FOXK1* and *DLC1* knockdown (KD) cells. Mechanistically, DLC1 was essential for FOXK1 occupancy to the promoter region of *MMP9*, and both DLC1 and FOXK1 acted cooperatively to transactivate MMP9 expression for melanoma invasion and metastasis. Consistently, DLC1, FOXK1, and MMP9 exhibited a high correlation of expression in melanoma patients’ samples and cell lines. Our results provide a new paradigm of the underlying mechanism by which a well-known tumor-suppressor DLC1 functions in the nucleus as an oncogene in melanoma.

## Results

### Elevated expression of *DLC1* in melanoma

**Fig. 1:**
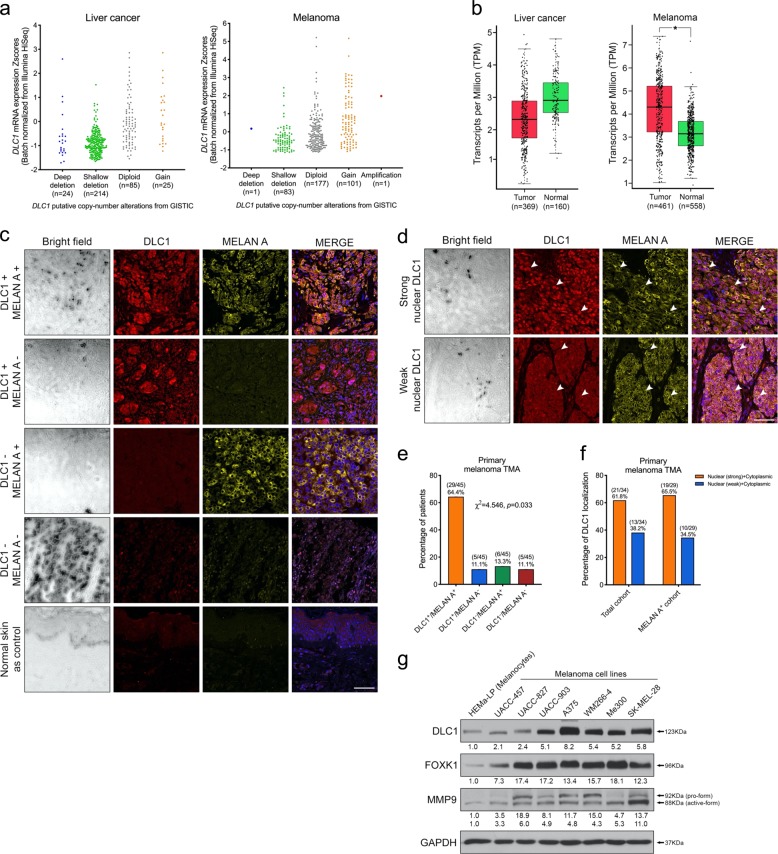
DLC1 expression in human melanomas and melanoma cell lines. **a** DLC1 copy-number variation analysis of liver cancer (*n* = 372) and melanoma (*n* = 363) cases from the TCGA database. Each dot represents data from one patient. **b** RNA-sequencing analysis of *DLC1* mRNA levels in liver cancer (*n* = 369) and melanoma (*n* = 461) samples compared with normal liver (*n* = 160) and skin (*n* = 558) tissues, respectively, from the TCGA database. *p < 0.05, by Student’s *t* test. **c**, **d** Immunofluorescence of DLC1 and MELAN A in 45 primary melanoma specimens in a tissue microarray (TMA). White arrowheads indicate melanoma cells with overlapping expression of nuclear DLC1 and cytoplasmic MELAN A. **e** Percentage of cases with DLC1^+^/MELAN A^+^, DLC1^+^/MELAN A^−^, DLC1^−^/MELAN A^+^, and DLC1^−^/MELAN A^−^ expression^.^ Chi-squared test, *p* = 0.033. **f** Percentage of cases with nuclear DLC1 (strong) + cytoplasmic and nuclear DLC1 (weak) + cytoplasmic in the total cohort and MELAN A^+^ cohort. **g** Western blot detection of DLC1, FOXK1. and MMP9 expression levels in normal melanocytes (HEMa-LP) and a panel of melanoma cell lines. GAPDH served as a loading control. The signal intensities of protein bands are shown in arbitrary units after normalization with GAPDH. Scale bar: 100 µM.

Examining the allelic alteration patterns of the *DLC1* locus revealed a larger portion of samples from melanoma patients with gain in copy number of *DLC1* than that of liver cancer patients (Fig. 1a). Analysis of the TCGA dataset showed higher levels of *DLC1* transcripts in cutaneous melanoma (*n* = 461) than normal tissue samples (*n* = 558) (Fig. 1b and Supplementary Fig. 1a). Consistent with its tumor-suppressor function, deep (6.9%) and shallow (61.5%) deletion of the *DLC1* gene were more frequently detected in human hepatocellular carcinoma than that of melanoma (deep, 0.28%; shallow, 22.9%) that correlated with lower *DLC1* mRNA expression levels among liver tumor samples (Fig. 1a, b) [26]. To further examine DLC1 protein expression in melanoma tissue samples, we performed immunofluorescence in a melanoma tissue microarray composed of 45 cores of primary cutaneous melanomas. We found DLC1 and a melanoma marker MELAN A exhibited high correlation of their expression (χ^2^ = 4.546, *p* < 0.033) in a large percentage of melanomas (29/45; 64.4%) compared with other specimens expressing either DLC1 (5/45; 11.1%) or MELAN A alone (6/45; 13.3%) and neither of them (5/45; 11.1%) (Fig. 1c, e). In addition to the detection of cytoplasmic DLC1 in all samples analyzed, we also observed strong (21/34; 61.8%) and weak (13/34; 38.2%) nuclear DLC1 expression in melanoma cells. Most of the melanomas showing strong nuclear DLC1 were MELAN A positive (19/29; 65.5%), whereas a small portion of MELAN A^+^ cells exhibited weak nuclear DLC1 (10/29; 34.5%) (Fig. 1d, f). These staining results are consistent with previous findings showing strong nuclear DLC1 staining in most of the primary melanomas (56%) from tissue microarrays [17]. Furthermore, DLC1 was expressed at higher levels in a panel of malignant melanoma cell lines than epidermal melanocytes (Fig. 1g). These expression analyses suggest that elevated levels and strong nuclear localization of DLC1 are associated with cutaneous melanomas.

### Nuclear DLC1 promotes melanoma growth and invasiveness in a RhoGAP-independent manner

**Fig. 2:**
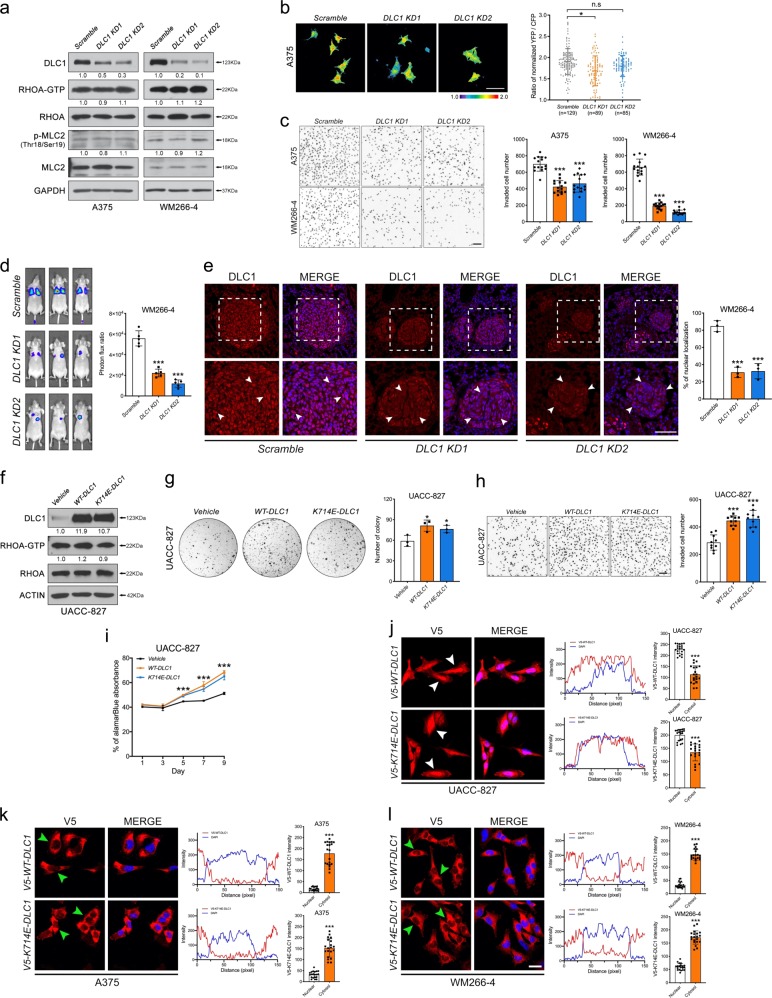
DLC1 promotes melanoma growth and metastasis in a RhoGAP-independent manner. **a** Western blots showing the levels of active RHOA-GTP and its downstream effector in *DLC1 KD* cells. **b** Measurement of RHOA-GTP activity by RHOA-FRET biosensor in A375 cells transduced with scrambled control (*n* = 129), *DLC1* KD1 (*n* = 89), or KD2 (*n* = 85). The activity of RHOA-GTP was quantified as the ratio of YFP to CFP and significance *p < 0.05, was calculated by one-way ANOVA. Data represented the mean ± SD. Scale bar: 50 µM. **c** Transwell invasion assay of A375 (*n* = 10) and WM266-4 cells (*n* = 10) subjected to the indicated treatment. Scale bar: 100 µM. **d** Bioluminescence images of lung-colonized cancer cells in NOD/SCID mice (*n* = 5 per treatment). Graph showing quantification of bioluminescence signal intensities. **e** Immunostaining of DLC1 expression on cross-sections of lung nodules derived from NOD/SCID mice subjected to the indicated treatments. White arrows indicate nuclear DLC1 staining. The magnified areas are marked with dashed boxes. Graph showing the percentage of cells with nuclear DLC1 in each treatment. **f** Immunoblots to detect ectopic WT-DLC1, K714E-DLC1, and active RHOA-GTP in the UACC-827 melanoma cell line. Actin served as a loading control. **g** Clonogenic (*n* = 3), **h** transwell invasion (*n* = 10), and **i** alamarBlue (*n* = 6) of UACC-827 cells transduced with the indicated constructs. **j**–**l** Immunostaining showing the detection of ectopic V5-WT-DLC1 and V5-K714E-DLC1 expression in UACC-827, A375, and WM266-4 melanoma cells. White and green arrowheads indicate the nuclear and cytoplasmic localization of ectopic V5-WT-DLC1 and V5-K714E-DLC1, respectively. Nuclei were counterstained with DAPI. Line scan analysis showing the intensities of nuclear and cytoplasmic DLC1 staining in cells treated with the indicated constructs (*n* = 20 per treatment). Scale bar: 50 µM. *p < 0.05, **p < 0.01, ***p < 0.001, by one-way ANOVA. Data represented the mean ± SD.

To examine whether DLC1 has a role in regulating melanoma growth and metastasis, we performed gene KD by designing two different shRNA lentiviral constructs to stably silence *DLC1* transcripts (*DLC1 KD1* and *DLC1 KD2*) in BRAF-mutated A375 and WM266-4 cell lines, as they showed high levels of DLC1 expression (Fig. 1g). Both shRNA constructs significantly reduced DLC1 protein levels (Fig. 2a). However, *DLC1 KD1* and *KD2* did not increase the levels of active RHOA-GTP and its downstream effector, phosphorylated myosin light chain 2 (p-MLC2) in melanoma cells (Fig. 2a, b), whereas elevation of RHOA activity was detected in *DLC1 KD* DU145 prostate cancer cells compared with the scrambled control (Supplementary Fig. 1b) consistent with previous findings [27, 28]. We then analyzed the effects of *DLC1 KD1* and *KD2* on invasion, tumorigenicity, and cell proliferation using transwell assays, colony formation, alamarBlue, and EdU incorporation assays, respectively. Both *DLC1 KD1* and *KD2* resulted in a marked reduction of invasive capacity, colony formation, and cell proliferation (Fig. 2c and Supplementary Fig. 1c–e). We further validated the effects of *DLC1 KD* on tumor growth in vivo, using xenograft models in mice. To this end, *DLC1*-depleted A375 melanoma cells were implanted subcutaneously in immunodeficient mice. After 4 weeks of injection, *DLC1 KD* cells caused a marked reduction in the size and weight of tumors formed compared with control (Supplementary Fig. 1f). To evaluate the role of DLC1 in melanoma metastasis, we performed lung colonization assay by injecting scrambled control, *DLC1 KD1* and *KD2* cells into the tail vein of NOD/SCID mice. The results showed that cells treated with the scrambled control displayed lung colonization 6 weeks post injection, whereas a striking reduction of the metastatic capacity of *DLC1*-depleted cells to the lung was observed (Fig. 2d and Supplementary Fig. 1g). Besides, most of the cells expressed nuclear DLC1 in the control sections of pulmonary nodules, whereas a marked reduction in the number of nuclear DLC1-expressing cells were observed in sections of *DLC1*-depleted lung nodules (Fig. 2e), confirming the cellular origin of cancer. Altogether, these data indicate that DLC1 is required to promote the invasiveness, tumorigenicity, and proliferation of melanoma cells in a RHOA-independent manner.

To further investigate whether the observed increase in *DLC1* expression levels in cutaneous melanomas functionally promotes their progression, we overexpressed (OE) wild-type DLC1 (WT-DLC1) and K714E-DLC1 (RhoGAP deficient mutant) [22] in A375 and WM266-4 melanoma cells (Supplementary Fig. 2a). We first analyzed their effects on RHOA activity. While WT-DLC1 but not K714E-DLC1 reduced the levels of active RhoA-GTP in DU145 prostate cancer cells as shown previously (Supplementary Fig. 1b) [27, 28], the levels of RhoA-GTP and p-MLC2 remained unaltered in melanoma cells treated with either WT-DLC1 or K714E-DLC1 (Supplementary Fig. 2a, b). Moreover, enforced expression of WT-DLC1 or K714E-DLC1 did not further enhance colony formation, proliferation, and invasiveness of melanoma cells (Supplementary Fig. 2c–f), whereas WT-DLC1 but not K714E-DLC1 inhibited colony formation, proliferation, and invasion of DU145 cells consistent with the tumor-suppressor properties of DLC1 acting through its RhoGAP activity in prostate cancer cells (Supplementary Fig. 2g–i) [27]. Conversely, OE of WT-DLC1 or K714E-DLC1 in low DLC1-expressing UACC-827 melanoma cells significantly promoted colony formation, invasion, and proliferation compared with vehicle control (Figs. 1g and 2f–i). However, both treatments did not alter RHOA activity (Fig. 2f). Intriguingly, by immunofluorescence, most ectopic WT-DLC1 and K714E-DLC1 proteins were detected in the nucleus of UACC-827 cells (Fig. 2j), whereas they were predominantly localized in the cytosol of high DLC1-expressing A375 and WM266-4 cells (Fig. 2k, l). These observations suggest that nuclear-localized DLC1 confers oncogenic properties in low DLC1-expressing melanoma cells. Indeed, ectopic WT-DLC1 was localized in the nucleus of *DLC1*-depleted A375 and WM266-4 cells (Supplementary Fig. 3a). Importantly, both WT-DLC1 and K714E-DLC1 were able to restore tumor growth from *DLC1*-KD A375 cells to a greater extent without altering RHOA activity (Supplementary Fig. 3b, c), further confirming the oncogenic role of nuclear DLC1 independent of RhoGAP. Domain mapping studies using a series of DLC1 truncated mutants with distinct subcellular localization revealed that constructs carrying both RhoGAP-START domains (residues 636–1091) and RhoGAP domain alone (residues 636–853) but not START domain alone (residues 878–1091) were capable of restoring the invasive behavior of *DLC1* KD cells even though they were all predominantly localized to the nucleus (Supplementary Fig. 4a–c), suggesting that oncogenic function of nuclear WT-DLC1 could be mediated through its RhoGAP domain. To further examine whether cytoplasmic DLC1 is required for oncogenesis, we took advantage of a dominant-negative form of DLC1 (DN-DLC1), which was predominantly localized in the cytoplasm of UACC-827 cells (Supplementary Fig. 4b, d) and able to antagonize endogenous and ectopic WT-DLC1 function on RHOA-GTP hydrolysis in DU145 cells (Supplementary Fig. 4e), consistent with previous findings in chick embryos [16]. However, cytoplasmic DN-DLC1 was ineffective in inhibiting melanoma invasion, antagonizing the oncogenic functions of ectopic WT-DLC1 and K714E-DLC1 in UACC-827 cells (Supplementary Fig. 4f), and restoring migration defects of *DLC1 KD* A375 and WM266-4 cells (Supplementary Fig. 4g, h), ruling out the oncogenic role of cytoplasmic DLC1. Taken together, our data indicate that low DLC1-expressing cells permit exogenous WT-DLC1 to be translocated into the nucleus to orchestrate the oncogenic events. Thus, the lack of oncogenic effects by forced expression of WT-DLC1 or K714E-DLC1 in high DLC1-expressing A375 and WM266-4 cells could be due to limited availability of cofactors that might be required for translocating exogenous DLC1 into the nucleus, implying that DLC1 may function with cofactors in the nucleus to promote melanoma growth and invasion in a RhoGAP-independent manner.

### FOXK1 promotes DLC1 nuclear translocation and retention through protein-protein interaction

**Fig. 3:**
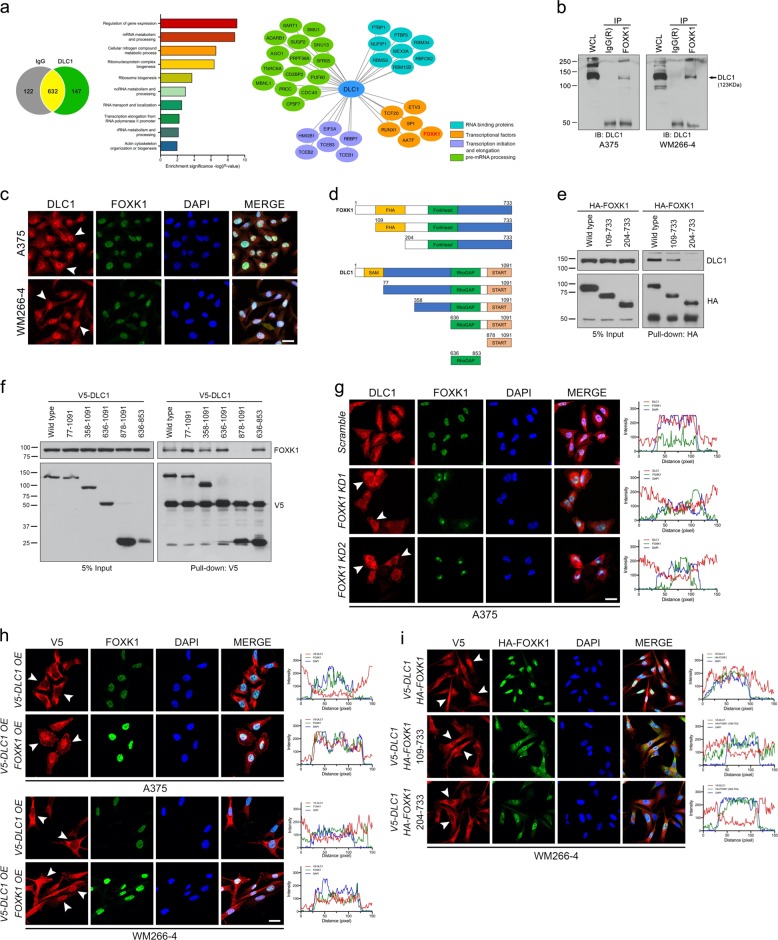
FOXK1 is DLC1-associated protein and promotes DLC1 nuclear translocation and retention. **a** The Venn diagram, GO term analysis, and interactome of DLC1-associated factors in A375 cells identified by mass spectrometry. IgG isotype served as a negative control. **b** Co-immunoprecipitation (Co-IP) showing the interaction between endogenous FOXK1 and DLC1 in A375 and WM266-4 cells. **c** Immunostaining showing the subcellular localization of endogenous DLC1 and FOXK1. White arrowheads indicate nuclear DLC1. **d** Schematic of full-length DLC1 and FOXK1 protein domains and their truncated constructs. **e** Co-IP analysis to determine the regions of FOXK1 required for interacting with DLC1. **f** Co-IP analysis to determine the region of DLC1 for interacting with FOXK1. **g** Immunostaining and line scan analysis showing the translocation of endogenous DLC1 from the nucleus to the cytosol in *FOXK1 KD* cells (white arrowheads) compared with predominant nuclear localization of DLC1 in the control. **h** Immunostaining and line scan analysis showing the cytoplasmic localization of ectopic V5-DLC1 in melanoma cells, whereas FOXK1 overexpression (OE) induces the translocation of ectopic V5-DLC1 from the cytoplasm to the nucleus. **i** Immunostaining and line-scanning analysis showing the ability of full-length HA-FOXK1 and to a lesser extent of the truncated FOXK1 (residues 109–733) but not the deletion mutant (residues 204–733) to induce the translocation of ectopic V5-DLC1 into the nucleus. DAPI stains cell nuclei. Scale bar: 50 µM.

To identify nuclear cofactors of DLC1 that may unravel the mechanisms by which DLC1 promotes melanoma progression, we performed immunoprecipitation for endogenous DLC1 in A375 cell lysates followed by mass spectrometry analysis. We revealed 147 unique proteins enriched by DLC1 and the majority of them were nuclear factors involved in mRNA processing and metabolism, as well as regulation of gene expression, compared with less abundant cytoplasmic proteins that contribute to actin cytoskeleton organization or biogenesis (Fig. 3a and Supplementary Table 1). We particularly focused on the interactome network of nuclear factors related to transcriptional regulation. Among them, we selected FOXK1 transcription factor (Fig. 3a), which belongs to the Forkhead family of the winged-helix DNA binding domain because it is expressed in migratory NC cells of *Xenopus* embryos [29] and has been shown to promote invasion and metastasis of various cancer types [30–32]. Immunoprecipitation studies in both A375 and WM266-4 cell lines in which endogenous levels of DLC1 and FOXK1 expression were high confirmed their interaction (Figs. 1g and 3b). Besides, immunofluorescence staining showed predominant nuclear localization of endogenous DLC1 and FOXK1 in melanoma cells (Fig. 3c), suggesting that DLC1 is likely to associate with FOXK1 in the nucleus. In agreement with this, the RhoGAP (residues 636–853) but not the START domain alone (residues 878–1091) was required for interacting with FOXK1 (Fig. 3d, f, and Supplementary Fig. 4a, b). Conversely, the Forkhead-associated (FHA) (residues 109–204) domain of FOXK1 was partly required for associating with DLC1, whereas no association of truncated FOXK1 (residues 204–733) with DLC1 was detected (Fig. 3d, e), implying that both the N-terminal region (residues 1–109) and the FHA domain of FOXK1 are crucial for interacting with DLC1. GST pull-down assay further confirmed a direct interaction between DLC1 and FOXK1 (Supplementary Fig. 5a). Previous studies showed that FOXK1 promoted disheveled protein nuclear translocation [33], raising the possibility that FOXK1 could regulate DLC1 nuclear localization. Indeed, endogenous DLC1 was found to be localized in the cytoplasm of *FOXK1 KD1* and *KD2* melanoma cells compared with its nuclear localization in the scrambled control (Fig. 3g and Supplementary Fig. 5b), suggesting that FOXK1 is required for retaining DLC1 in the nucleus. While ectopic DLC1 protein mostly resided in the cytoplasm, OE of DLC1 and FOXK1 together enhanced the nuclear localization of ectopic DLC1 (Fig. 3h), further indicating an essential requirement for FOXK1 to promote DLC1 nuclear translocation. Both the wild-type and to a lesser extent of the truncated version of FOXK1 (residues 109–733) but not a deletion mutant (residues 204–733) were able to promote DLC1 nuclear localization that coincided with their capability to associate with DLC1 (Fig. 3e, i, and Supplementary Fig. 5c). These data suggest that the RhoGAP domain of DLC1 and the N-terminal region including the FHA motif of FOXK1 (residues 1–204) are required for their interaction, which is essential for nuclear translocation and retention of DLC1.

### FOXK1 is oncogenic in melanoma

**Fig. 4:**
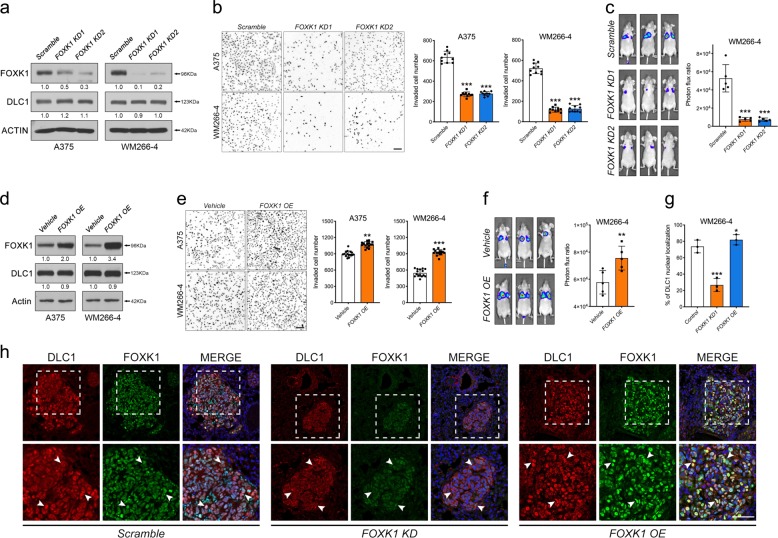
FOXK1 promotes melanoma growth and metastasis. **a**, **d** Western blots to detect endogenous FOXK1 and DLC1 expression in cells treated with the indicated constructs. Actin served as a loading control. **b**, **e** Transwell invasion (*n* = 10) of A375 and WM266-4 cells transduced with the indicated constructs. Scale bars: 100 μM**. c**, **f** Bioluminescence images of lung-colonized tumor cells in NOD/SCID mice (*n* = 5 per treatment) which were injected with the indicated constructs into the tail vein. Graph showing quantification of the bioluminescence signal intensities. **g**, **h** Statistical analysis of DLC1 nuclear localization and immunostaining of DLC1 and FOXK1 on cross sections of lung nodules from NOD/SCID mice subjected to the indicated treatments. The magnified areas are marked with dashed boxes. White arrowheads indicate subcellular localization of DLC1 in different treatments. *p < 0.05, **p < 0.01, ***p < 0.001, by one-way ANOVA. Data represented the mean ± SD.

We then analyzed the role of FOXK1 in melanoma progression. To this end, we designed two shRNA to deplete *FOXK1* (*FOXK1 KD1* and *KD2*) in A375 and WM266-4 cells (Fig. 4a), as they expressed high levels of FOXK1 expression compared with normal melanocytes (Fig. 1g). Cells treated with *FOXK1 KD1* and *KD2* exhibited a marked reduction in invasive capacities, proliferation, and the number of colonies formed, compared with scrambled control (Fig. 4b and Supplementary Fig. 6a, b). Consistently, tail vein injection and subcutaneous implantation of *FOXK1 KD* cells into immunodeficient mice led to a reduction in the number of lung nodules and smaller tumors respectively, when compared with scrambled control (Fig. 4c and Supplementary Fig. 6c, d). Conversely, OE of FOXK1 (FOXK1 OE) promoted melanoma tumorigenicity, growth, and metastasis both in vitro and in vivo (Fig. 4d–f and Supplementary Fig. 6e–h). By immunostaining, we detected an increase in the number of tumor cells expressing nuclear DLC1 on cross sections of lung nodules treated with FOXK1 OE compared with control, whereas DLC1 was barely observed in the nucleus of *FOXK1 KD* cells (Fig. 4g, h), further consolidating our in vitro observations that FOXK1 is sufficient and required for nuclear translocation and retention of DLC1, respectively. Importantly, DLC1 expression levels remained unaltered in both *FOXK1*-depleted and FOXK1 OE melanoma cells (Fig. 4a, d), indicating that DLC1 expression is not regulated by FOXK1. Similarly, FOXK1 expression remained unaltered in *DLC1 KD* cells, confirming their cofactor relationship (Supplementary Fig. 6i). Altogether, these results suggest that FOXK1 functions as an oncogene in melanoma progression likely through partnering with DLC1 in the nucleus.

### DLC1 regulates FOXK1-binding capacity to the *MMP9* promoter region

**Fig. 5:**
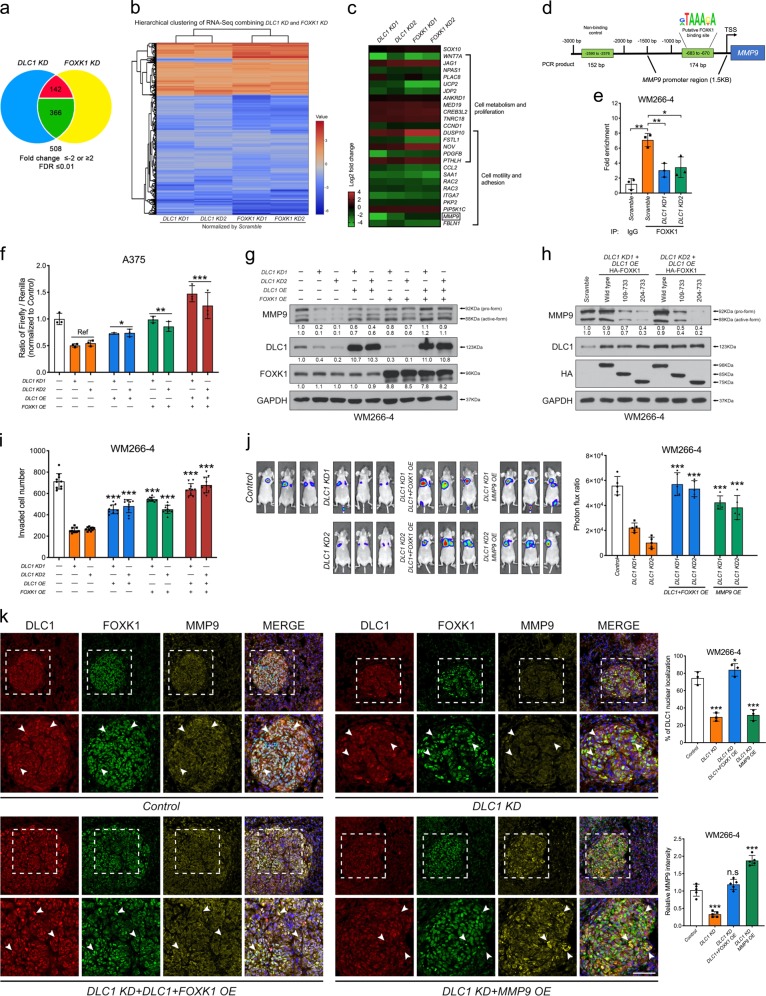
DLC1 and FOXK1 cooperatively transactivate MMP9 expression and promote melanoma invasion and metastasis. **a** Venn diagram illustrating the number of genes commonly upregulated (red; *n* = 142) and downregulated (Green; *n* = 366) in both *DLC1 KD* and *FOXK1 KD* cells. **b** Hierarchical clustering of RNA-sequencing data from *DLC1 KD* and *FOXK1 KD* normalized with scrambled control. **c** Heat maps of selected candidates under the functional categories of metabolism, proliferation, motility, and adhesion. **d** Schematic of *MMP9* promoter region harboring *FOKX1* binding motif. The region without the FOKX1 binding motif served as a negative control. **e** Determination of FOXK1-binding capacity on the *MMP9* promoter region in the indicated treatments by ChIP-qPCR compared with scrambled and IgG controls. Samples were assayed in triplicate. **f** A375 cells were transfected with a 1.5 kb *MMP9* promoter-driven luciferase reporter construct plus *Renilla* for normalization of transfection efficiency and the indicated treatments. *DLC1* KD was set as a reference for statistical analysis. **g, h** Immunoblots revealing different degrees of restoring MMP9 expression in melanoma cells treated with the indicated constructs. **i** Transwell invasion assay showing the different degrees of restoring invasiveness of cells treated with the indicated constructs (*n* = 10 per treatment). **j** Bioluminescence images of lung-colonized tumor cells in NOD/SCID mice (*n* = 5 per treatment) treated with the indicated constructs. Graph showing quantification of bioluminescence signal intensities. **k** Immunostaining of DLC1, FOXK1, and MMP9 expression on cross sections of lung nodules from NOD/SCID mice. White arrowheads indicate the nuclear localization of DLC1. The magnified areas are marked with dashed boxes. Graphs showing statistical analysis of DLC1 nuclear localization and relative MMP9 intensity on tumor sections treated with the indicated constructs. *p < 0.05, **p < 0.01, ***p < 0.001, by one-way ANOVA. Data represented the mean ± SD.

To further unravel the molecular mechanisms by which DLC1 associates with FOXK1 to regulate melanoma metastasis, we undertook RNA-seq analysis of both *DLC1 KD* and *FOXK1 KD* A375 cells to identify changes in gene expression compared with scrambled control (Datasets 1 and 2). Hierarchical clustering of both *KD* datasets normalized by control revealed a significant portion of commonly downregulated and upregulated genes (Fig. 5a, b), suggesting that DLC1 and FOXK1 act together to regulate the same cellular processes. Gene ontology (GO) analysis of regulated targets showed enrichment in categories related to cell metabolism, proliferation, adhesion, motility, and among others (Fig. 5c). Among the commonly downregulated targets involved in cancer invasion and metastasis, we selected secreted MMP9 for further studies based on previous reports that it is required for both NC cell migration and melanoma invasiveness via the degradation of extracellular matrix [34–36]. Consistent with its expression change in RNA-seq, we confirmed the downregulation of MMP9 at the mRNA and protein levels in both *DLC1 KD* and *FOXK1 KD* melanoma cells (Supplementary Fig. 7a–d). To investigate whether MMP9 expression was directly regulated by FOXK1, we identified a potential FOXK1-binding site (−683 bp to −670 bp) within the 1.5 kb *MMP9* promoter region based on the conserved sequence motif (WRTAAAAYA) recognized by FOXK1 in previous studies (Fig. 5d) [37, 38]. Chromatin immunoprecipitation (ChIP) assay confirmed a higher binding affinity of FOXK1 for the consensus motif within the *MMP9* promoter in melanoma cells compared with IgG control, whereas it did not bind to another DNA fragment without the motif (Fig. 5e and Supplementary Fig. 7e), indicating the specificity of the binding. Conversely, the ability of FOXK1 to bind to this motif was significantly reduced in *DLC1 KD* cells (Fig. 5e and Supplementary Fig. 7e), suggesting that DLC1 is required for FOXK1 occupancy to the promoter region of *MMP9*. In agreement with this, *DLC1 KD* reduced *FOXK1*-promoter-driven luciferase reporter activity, which was restored to a greater degree by FOXK1 OE than DLC1 OE. Combined expression of DLC1 and FOXK1 further augmented the reporter activity (Fig. 5f), suggesting cooperative transactivation of the *MMP9* promoter. To further investigate whether MMP9 could mediate the role of DLC1 and/or FOXK1 in melanoma invasion, cells treated with *DLC1 KD* or *FOXK1 KD* together with MMP9 overexpression (MMP9 OE) were subjected to transwell assay. The results showed a complete restoration of invasive potential for *DLC1 KD* + MMP9 OE cells (Supplementary Fig. 7f, g). In contrast, cells expressing *FOXK1 KD* + MMP9 OE (77.9% in A375 and 75.78% in WM266-4) displayed a partial rescue of invasion compared with *FOXK1 KD* alone (43.1% in A375 and 23.4% in WM266-4) (Supplementary Fig. 7h, i). Taken together, the data suggest that DLC1 and FOXK1 function in a cooperative manner to transactivate *MMP9* promoter activity as well as MMP9 functions downstream of DLC1 and FOXK1 to promote melanoma invasion in vitro.

### DLC1–FOXK1 complex acts in a cooperative manner to activate MMP9 expression for promoting melanoma metastasis in vitro and in vivo

To further substantiate the cooperative action of the DLC1–FOXK1 complex in regulating MMP9 expression and promoting melanoma invasiveness, we examined the ability of DLC1 OE, FOXK1 OE, and both to restore MMP9 expression and melanoma invasion in *DLC1 KD* cells. While FOXK1 OE exhibited a greater degree of restoration in MMP9 expression than DLC1 OE, combined OE resulted in further augmenting MMP9 induction (Fig. 5g and Supplementary Fig. 7j). Consistent with reduced ability of truncated FOXK1 carrying FHA motif (109-733) to interact with DLC1, the degree of MMP9 activation by both constructs was lesser extent compared with OE of WT-FOXK1 and WT-DLC1. Besides, the expression levels of MMP9 remained low in *DLC1 KD* cells overexpressing both DLC1 and truncated versions of FOXK1 (residues 204–733), which exhibited a lack of interaction with DLC1 (Figs. 3e and 5h and Supplementary Fig. 7k). These results suggest that the interaction between DLC1 and FOXK1 is crucial for cooperative transactivation of MMP9 expression. Similarly, OE of both DLC1 and FOXK1 also further enhanced the invasiveness of *DLC1 KD* melanoma cells than OE of each factor in vitro (Fig. 5i and Supplementary Fig. 7l, m). In agreement with this, the lung colonization capacity of *DLC1*-depleted cells that were OE with DLC1 and FOXK1 was markedly restored, whereas *DLC1 KD* cells gave rise to smaller and reduced number of pulmonary nodules compared with control (Fig. 5j, k). Consistently, we detected an increased number of cells expressing nuclear DLC1 and restoration of MMP9 expression on cross sections of pulmonary nodules from the combined expression of DLC1 and FOXK1 compared with low levels of nuclear DLC1 and MMP9 expression in *DLC1* KD cells (Fig. 5k). Accordingly, MMP9 was sufficient to restore the number of lung nodules formed in mice treated with *DLC1 KD* cells (Fig. 5j, k). These results further confirm that DLC1 functions with FOXK1 in a cooperative manner to activate MMP9 expression for promoting melanoma metastasis.

**Fig. 6:**
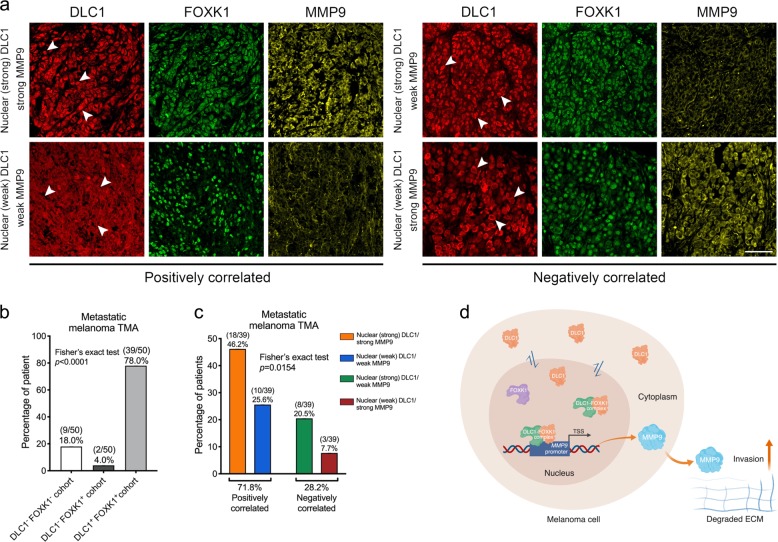
Correlation of DLC1, FOXK1, and MMP9 expression in metastatic melanoma tissue microarray. **a** Immunofluorescence showing DLC1, FOXK1, and MMP9 expression on representative tissue sections from the different categorization of metastatic melanomas (*n* = 50). White arrowheads indicate melanoma cells with strong or weak DLC1 staining in the nuclei. Scale bar: 100 µM. **b** Percentage of cases showing expression of neither DLC1 and FOXK1, FOXK1 alone, and both together. Fisher’s exact test *p* < 0.0001 to indicate highly correlated expression between DLC1 and FOXK1 in 39 out of 50 cases. **c** Percentage of cases with nuclear (strong) DLC1/strong MMP9, nuclear (weak) DLC1/weak MMP9, nuclear (strong) DLC1/weak MMP9, and nuclear (weak) DLC1/strong MMP9. Fisher’s exact test *p* = 0.0154 indicates a positive correlation of expression levels between nuclear DLC1 and MMP9. **d** Schematic diagram showing nuclear DLC1 to recruit FOXK1 for cooperative activation of MMP9 expression, which promotes melanoma invasion and metastasis via degradation of extracellular matrix (ECM).

To correlate the clinical relevance of DLC1, FOXK1, and MMP9 expression, we analyzed their expression in a melanoma tissue microarray composed of 50 cores of metastatic melanomas (Fig. 6a–c). We found a highly correlated expression between DLC1 and FOXK1 in the majority of metastatic melanomas (39/50, 78%; *p* < 0.0001), in which the levels of nuclear DLC1 exhibited positive correlation with MMP9 expression (18/39, 46.2% with strong expression in both DLC1/MMP9; 10/39, 25.6% with weak expression in both DLC1/MMP9; *p* = 0.0154) (Fig. 6a–c). These results suggest the importance of the DLC1 dosage requirement for determining the level of MMP9 expression likely through the regulation of FOXK1-binding capacity to *MMP9* regulatory regions. Also, we also detected higher levels of expression of the three proteins in all melanoma cell lines than epidermal melanocytes (Fig. 1g). The negative correlation between DLC1 and MMP9 expression in a relatively low percentage of cases could be due to the great heterogeneity between tumor samples that may harbor other genetic and/or epigenetic factors to alter the regulatory relationship between DLC1/FOXK1 and MMP9 (Fig. 6a, c). Nevertheless, our data demonstrate that nuclear DLC1 is clinically associated with FOXK1 to promote melanoma invasion and metastasis through cooperative activation of MMP9 expression (Fig. 6d).

## Discussion

Elucidating the molecular mechanisms that govern melanoma invasion and metastasis remain crucial to understand the disease progression and development of therapeutic strategies. Here, we demonstrate that DLC1, which is known to function as a tumor suppressor in various cancer types, plays oncogenic roles in melanoma, through the association of transcription factor FOXK1 in the nucleus for cooperative activation of MMP9 expression to promote invasion and metastasis.

One of the most unanticipated findings in this study is the increased expression of DLC1 in melanoma patient samples and cell lines compared with normal tissues and melanocytes, respectively. Conversely, downregulation of DLC1 via genetic and epigenetic mechanisms occurs frequently in a variety of cancers, including liver cancer, lung cancer, colorectal cancer, prostate cancer, and breast cancer [26, 39–41]. Whether distinct regulatory mechanisms contribute to the increased expression of DLC1 in melanoma remain to be elucidated. Since a substantial number of melanoma patients had *DLC1* copy-number gain, this allelic alteration may be a major factor driving the elevation of *DLC1* expression in this tumor type.

Our findings detected strong and weak levels of nuclear DLC1 expression in the majority of primary and metastatic melanomas, whereas previous studies revealed a marked reduction in the percentage of samples with nuclear DLC1 expression in metastatic melanomas compared with primary melanomas [17]. This discrepancy could be due to differences in staining methods with higher sensitivity immunofluorescence than immunoperoxidase, which might not be able to detect low levels of nuclear DLC1 expression. Nevertheless, our loss- and gain-of-function assays in vitro and in vivo provide strong evidence to support an oncogenic role of DLC1 in determining the tumorigenicity and metastatic behavior of melanoma. This is in sharp contrast to previous findings in other cancer types, in which *DLC1 KD* promoted cell proliferation, tumor formation, and invasion, while DLC1 OE reversed these phenotypes [39–42]. The majority of these studies attributed the tumor-suppressor role of DLC1 to its RhoGAP domain, which inhibits the small GTPases RHOA/B/C and CDC42 [43]. Conversely, we did not observe significant alteration of RHOA activity and its downstream events in both DLC1 KD and OE melanoma cells. Besides, OE of RhoGAP deficient mutant (K714E), which was shown to abolish the suppressive function of DLC1 in liver cancer [22], exhibited a similar oncogenic activity to WT-DLC1 in promoting melanoma growth and invasion, further supporting the notion that DLC1 functions in a RhoGAP-independent manner to orchestrate the oncogenic events. It remains unclear as to why DLC1 OE failed to inhibit RHOA activity in melanoma cells. Previous studies showed that activated protein kinase C and protein kinase D phosphorylated DLC1 and promoted the association of DLC1 with 14-3-3 adapter protein, leading to the blockade of RhoGAP activity [44]. Besides, a recent report revealed that AKT-mediated phosphorylation of the serine residues, located N-terminal to the DLC1 RhoGAP domain, greatly attenuated its RhoGAP and tumor-suppressor activities [45]. Based on these studies, it is, therefore, tempting to speculate that phosphorylation could be one of the regulatory mechanisms to inhibit the RhoGAP activity of exogenous DLC1 expression provided that protein kinases are not in limiting amount. In addition, the lack of increased RHOA activity in *DLC1 KD* melanoma cells could be due to predominant nuclear localization of endogenous DLC1, which was shown to be less efficient in mediating RHOA inhibition in other cancer cell types compared with cytoplasmic DLC1 [25].

Previous studies documented the ability of DLC1 to undergo nuclear translocation in different cellular contexts. Yuan et al. first reported that nuclear-localized DLC1 could induce apoptosis in non-small cell lung cancer cell lines [46]. Chan et al. demonstrated the abrogated tumor-suppressor activity of nuclear DLC1 compared with cytoplasmic DLC1 in hepatocellular carcinoma [25]. On the contrary, our findings revealed that ectopic WT-DLC1 in UACC-827 cells expressing low-level endogenous DLC1 (DLC1^LOW^) was preferentially localized in the nucleus that conferred oncogenic properties, whereas exogenous DLC1 was detected in the cytoplasm of A375 and WM266-4 cells expressing high levels of endogenous DLC1 (DLC1^HIGH^) without exerting tumorigenic effects. Mechanistically, our data demonstrated that FOXK1 as a cofactor of DLC1 was sufficient and required for nuclear translocation and retention of DLC1, respectively. Hence, differential responses to DLC1 OE in melanoma cell lines expressing distinct levels of endogenous DLC1 could be due to the limited amount of FOXK1 available to translocate exogenous DLC1 into the nucleus of DLC1^HIGH^ cells, whereas FOXK1 is still in excess to promote nuclear translocation of ectopic DLC1 in DLC1^LOW^ cells. In agreement with this, WT-DLC1 OE but not cytoplasmic DN-DLC1 can restore the invasion of *DLC1*-depleted cells. Importantly, DN-DLC1 is not sufficient to promote the invasive behavior of DLC1^LOW^ cells like nuclear WT-DLC1, further ruling out the contribution of cytoplasmic DLC1 to govern melanoma growth and progression.

Our results identified MMP9 as one of the downstream targets to mediate the roles of DLC1 and FOXK1 in promoting melanoma cell invasion and metastasis. By ChIP, we showed that DLC1 was required for the efficient binding of FOXK1 to the MMP9 promoter region. We propose two explanations as to how a RhoGAP DLC1 could regulate FOXK1 occupancy on the promoter region of MMP9. First, the interaction between DLC1 and FOXK1 may alter FOXK1 protein conformation to promote its DNA binding affinity. Second, our DLC1-interactome database also identified epigenetic regulators that could modify the chromatin state of the MMP9 promoter region to allow ease of FOXK1 access to its binding motif for transcriptional regulation. Further studies are required to examine these possibilities. Nevertheless, the DLC1–FOXK1 complex further augmented the degree of transactivation of *MMP9* gene expression as well as melanoma invasion and metastasis. The regulatory relationship between nuclear DLC1–FOXK1 complex and MMP9 is further supported by a strong correlation of their expression in metastatic melanomas as well as a panel of melanoma cell lines.

In summary, this is the first report showing nuclear-localized DLC1 to function as an oncogene through FOXK1 recruitment to cooperatively transactivate MMP9 expression for promoting melanoma invasion and metastasis. Follow up study to elucidate the molecular mechanisms that confer DLC1 with oncogenic activity in melanoma but not in other cancer types will provide new insight into the context-dependent role of DLC1. At last, our results have broader implications of RhoGAP family proteins, which might involve in transcriptional regulation in different cellular contexts.

## Materials and methods

### Cell lines and in vitro functional assays

All cell lines were authenticated by cell profiling (AmpFISTR Identifier PCR Amplification kit, Life Technologies) and mycoplasma-free as verified by Faculty Core Facility, the University of Hong Kong. The human melanoma cell lines A375, WM266-4, UACC-457, UACC-827, UACC-903, Me300, SK-Mel-28, human epidermal melanocyte light-pigmented (HEMa-LP) and human prostate cancer cell line DU145 were cultured as described in Supplementary Materials and Methods.

Cell proliferation by alamarBlue assay (ThermoFisher), EdU incorporation staining by the Click-iT EdU Alexa Fluor 488 Imaging Kit (Thermo Fisher), the clonogenic assay, transwell invasion assay using cell culture insert (Falcon) and Matrigel (Corning), and fluorescence resonance energy transfer analysis to detect RHOA activity were conducted as described in Supplementary Materials and Methods.

### Plasmids and gene expression and measurement

Lentiviral constructs for gain- and loss-of-function analysis were packaged using the third-generation system (see Supplementary Materials, available online). Quantitative polymerase chain reaction (qPCR) and dual-luciferase reporter assays (Promega) were conducted as described in Supplementary Materials and Methods. The list of shRNA oligos and qPCR primers is shown in Supplementary Table 2.

### Immunoblotting, immunoprecipitation, RHOA activity, and GST pull-down assays

Co-immunoprecipitation using magnetic beads IP kit (Thermo Fisher), ChIP using Pierce Magnetic ChIP Kit (Thermo Fisher), RHOA activity assay (Cytoskeleton), and GST pull-down assay were conducted as described in Supplementary Materials and Methods. The list of primary antibodies for immunoblotting is shown in Supplementary Table 3.

### Tissue samples, immunostaining, and quantification

Tissue microarray of malignant melanoma (ME1002b) and metastatic melanoma (BCC38218) were purchased from US Biomax Inc. Detailed procedure for antigen retrieval, immunostaining with primary antibodies (Supplementary Table 3), and line-scanning quantification are described in Supplementary Materials and Methods. Images were captured using LSM 780 confocal system (Carl Zeiss) maintained by the University of Hong Kong Li Ka Shing of Medicine Faculty Core Facility and analyzed by ZEN software (Carl Zeiss).

### Mass spectrometry and gene expression profiling

GO enrichment analysis was performed using PANTHER for the unique protein interactors identified from the DLC1 pull-down assay. Interactions of the identified target proteins were further generated by Cytoscape software with a confidence cut off of 0.6. In the resulting protein interactome, proteins were presented as nodes that are connected by lines with the confidence level (0.6–0.9). Mass spectrometry identification of protein candidates pulled down by DLC1 and IgG control is shown in Supplementary Table 1.

For gene expression profiling, total RNA from melanoma cells with different treatments was isolated and purified. RNA quality and quantity were analyzed by Agilent 2100 Bioanalyzer at the Beijing Genome Institute (BGI, Shenzhen). SurePrint G3 Human Gene Expression v3 8 × 60 K Microarray Kit (Agilent Technologies) covering 26,083 Entrez genes was used for gene expression profiling analysis. Statistical significance of the expression data was determined using fold change. Hierarchical cluster analysis was performed using complete linkage and Euclidean distance as a measure of similarity. The RNA-sequencing data and list of differentially expressed genes between *DLC1* KD, *FOXK1* KD, and control have been deposited in Figshare (https://figshare.com/s/88763da1b1afd4104755).

### In vivo xenograft and pulmonary metastasis assay

For subcutaneous xenograft, a 100 µL single-cell suspension containing 1 × 10^6^ A375 cells with different treatments in plain DMEM was injected into the right flank of 6-week-old female BALB/c immunocompromised mice. Four weeks post injection, mice were sacrificed using CO_2_ and subcutaneous tumor tissues were excised for size and weight measurements. For pulmonary metastasis assay, a 100 µL single-cell suspension containing 1 × 10^6^ WM266-4 cells with different treatments in plain EMEM was injected into the tail vein of 5-week-old female NOD/SCID mice. Five weeks post injection, mice were anesthetized before intraperitoneal injection of 100 µL of sterile D-luciferin firefly potassium salt solution (30 mg/mL, Biovision). The bioluminescent signals in lung-colonized cancer cells were acquired for 4 min in vivo imaging using Xenogen IVIS 200 in the University of Hong Kong Li Ka Shing Faculty of Medicine Faculty Core Facility. Regions of interest were manually selected, and the results were quantified as the average radiance of photons emitted per second and area by using the Living Image software (Xenogen).

### TCGA data mining

*DLC1* copy-number variation together with RNA-Seq data was generated and quantified using cBioportal for Cancer Genomics (http://www.cbioportal.org), under the category of Skin Cutaneous Melanoma (TCGA, PanCancer Atlas). Dot plot was generated by Gene Expression Profiling Interactive Analysis (http://gepia.cancer-pku.cn) to compare the gene expression profile between different cancer types and respective normal tissues.

### Statistical analysis

All quantitative data were presented as the mean ± SD (SD = standard deviation of the mean value in each independent experiment) and at least three independent experiments were performed. Student’s *t* test and one-way analysis of variance (ANOVA) were used to determine the confidence levels for group comparison. Pearson’s chi-square test and Fisher’s exact test were used to determine the correlation of two variables. Statistical significance was determined if *p* < 0.05.

## Supplementary information

Supplementary Material

Dataset 1

Dataset 2
