# Supplementary material for: Nuclear DLC1 exerts oncogenic function through association with FOXK1 for cooperative activation of MMP9 expression in melanoma
Source: Oncogene. 2020 Mar 25;39(20):4061–76. doi: 10.1038/s41388-020-1274-8 (PMC7220869; doi:10.1038/s41388-020-1274-8)
Supplement: Supplementary file 1 — Supplementary Material [file 41388_2020_1274_MOESM1_ESM.pdf]

# Nuclear DLC1 exerts oncogenic function through association with FOXK1 for cooperative activation of MMP9 expression in melanoma

Xintao Yang, Feng Hu, Jessica Aijia Liu, Shan Yu, May Pui Lai Cheung, Xuelai Liu, Irene Oi-Lin Ng, Xin-Yuan Guan, Kelvin KW Wong, Rakesh Sharma, Hong Lok Lung, Yufei Jiao, Leo Tsz On Lee and Martin Cheung

Correspondence to: Martin Cheung, PhD, School of Biomedical Sciences, Li Ka Shing Faculty of Medicine, The University of Hong Kong, 21 Sassoon Road, Pokfulam, Hong Kong, China. Email: mcheung9@hku.hk

**This file contains:**

Supplementary Materials and Methods

Supplementary Figures 1-7

Supplementary Tables 1-3

**Supplementary Materials and Methods**

**Plasmids and reagents**

Human *DLC1* (NM_006094.4), *FOXK1* (NM_001037165.2) and *MMP9* (NM_004994.3) cDNA were synthesized based on RNA template extracted from A375 melanoma cells and cloned into lentiviral vector pLVX-EF1α-IRES-Puro (Clontech). *DLC1-K714E* mutant was generated from wild type *DLC1* by using the Q5 Site-Directed Mutagenesis Kit (New England BioLabs) according to the manufacturer's protocol. The shRNA oligos targeting human *DLC1* and *FOXK1* are shown in Supplementary Table 2. Lentiviral vector pLKO.1-TRC-Puro (Addgene) was used for expressing the shRNA oligos. The RHOA-FRET-Biosensor insert was amplified from the plasmid pTriEx-RHOA-WT Biosensor (gift from Dr. Louis Hodgson), then cloned into piggyBac-CAGK vector for electroporation.

For Western blot and immunostaining analysis, the primary antibodies used are listed in Supplementary Table 3.

**Cell culture**

Human melanoma cell lines A375, UACC-457, UACC-827 and human embryonic kidney cell line 293T were cultured in DMEM medium with high glucose (Life Technologies) supplemented with 10% fetal bovine serum (FBS) (BioSera) and 100U/ml penicillin-streptomycin (Life Technologies). Human melanoma cell line WM266-4 and prostate cancer cell line DU145 were obtained from ATCC and cultured in EMEM medium (Sigma) supplemented with 10% FBS and 100U/ml penicillin-streptomycin. Human epidermal melanocyte adult with light-pigment (HEMa-LP) was purchased from ThermoFisher and cultured in Medium-254 supplemented with HMGS-2. RPMI-1640 medium (Life Technologies) was used to culture UACC-903,

LAU-Me300 and SK-MEL-28 kindly provided by D Leung, the Hong Kong University of Science and Technology. Cell lines were authenticated by cell profiling (AmpFISTR Identifier PCR Amplification kit, Life Technologies) and verified as mycoplasma-free by Faculty Core Facility, the University of Hong Kong.

**Western blot and co-immunoprecipitation (Co-IP)**

Cells with different treatments were rinsed twice with cold phosphate-buffered saline (PBS) and lysed in RIPA buffer (150mM NaCl, 1mM EDTA, 1% NP40, 0.5% Sodium deoxycholate, 0.1% SDS, 50mM Tris-HCl, pH 7.5) supplemented with 1% protease and phosphatase inhibitor cocktail (ThermoFisher). For co-immunoprecipitation, cells were lysed with IP buffer (150 mM NaCl, 1 mM EDTA, 1% NP-40, 25 mM Tris-HCl, 5% Glycerol, pH 7.4). A antibodies against specific proteins or IgG control were mixed with same amount of cell lysates (antibody: lysates=1:1000) and incubated at 4°C overnight with agitation. Then 25uL of MS-compatible magnetic Protein A/G beads (ThermoFisher) was added to each treatment and incubated at 4°C for 2 hours. Following the washing protocol provided by manufacturer, pulled-down proteins were eluted by using 2X Laemmli protein sample buffer (Bio-Rad). Proteins were separated by SDS-PAGE using the Bio-Rad system under reducing condition. Membranes were blocked with 5% skimmed milk and probed with antibodies against corresponding proteins for overnight at 4°C and then incubated with horseradish peroxidase-conjugated goat anti-rabbit and rabbit anti-mouse (Dako) at room temperature for 1 hour. After incubation with ECL substrate (Advansta), blots were exposed to X-ray film (Super RX, FujiFilm) at different times to obtain the optimal intensity of the protein bands followed by analysis using ImageJ.

**Quantitative polymerase chain reaction (qPCR)**

Total RNA was extracted using MiniBEST Universal RNA Extraction Kit (Takara) and reverse transcribed for cDNA synthesis using PrimeScript RT Master Mix (Takara). All reactions including non-template controls were performed in triplicate on StepOnePlus Real-time PCR system (Applied Biosystem) using SYBR Premix Ex Taq II (Takara). Human *36B4* was used for normalization. The list of primers for detection of gene expression are shown in Supplementary Table 2.

**Chromatin immunoprecipitation (ChIP)**

A total of 2 × $10^6$ WM266-4 melanoma cells stably expressing *Scramble* control or *DLC1* silencing shRNA were crosslinked by 1% formaldehyde and lysed, then digested using 5.0μL diluted micrococcal nuclease according to the manufacturer's protocol (Pierce Magnetic ChIP Kit, ThermoFisher). Supernatant was collected and sonicated for 6 × 30 s in a BioruptorPico Sonicator (Diagenode). The target size of chromatin fragments ranging from 100 bp to 600 bp was confirmed by 2% agarose gel electrophoresis. Chromatin fragments were immunoprecipitated by using normal rabbit IgG control (ThermoFisher) or 4μg anti-FOXK1 antibody (ChIP grade, ab18196, Abcam) at 4°C overnight. 20uL of ChIP grade magnetic beads (ThermoFisher) was then added into the chromatin-antibody mixture and incubated at 4 °C for 2 hours. DNA fragments were then purified and reserve-crosslinked based on manufacturer's instruction, followed by 40 cycles of quantitative PCR. The PCR products were further visualized by 2.5% agarose gel electrophoresis. The primers used for amplification of fragments spanning FOXK1 binding motif on *MMP9* promoter are summarized in Supplementary Table 2. Data were analyzed and presented as the fold enrichment relative to IgG control.

**Dual-luciferase reporter assay**

Melanoma cells were transfected with pGL3-basic FireFly luciferase reporter vector (Promega) driven by human MMP9 proximal promoter (~ 1.5 kb) and Renilla luciferase reporter vector using PolyJet transfection reagent based on manufacturer's protocol. Cells were harvested and lysed 48 hours post-transfection. The cell lysate was measured by PerkinElmer Victor 3 Multi-label Plate Reader using Dual-Luciferase Reporter Assay System (Promega) according to the manufacturer's instructions. The luminescence signal of the Renilla luciferase reporter activity was used for normalization of FireFly luciferase reporter activity.

**Lentiviral production and transduction**

$7x10^6$ 293T cells in a 100mm culture dish with 90-100% confluence were co-transfected with a lentiviral expression vector, packaging plasmid psPAX.2 and envelope plasmid pMD2.G using PolyJet (SignaGen) according to the manufacturer's instruction. The cell culture medium containing the lentiviral particles was harvested 48- and 72-hour post transfection and filtered through a 0.22μm filter. $3x10^5$ melanoma cells were infected with lentivirus particles expressing cDNA and/or shRNA and cultured in the presence of 8μg/ml Polybrene (Sigma) for 24 hours. After 48 hours transduction, infected melanoma cells were screened in presence of 1μg/ml puromycin (Life Technologies) for 96 hours to generate stable cell lines.

**Colony formation assay**

Single cells ($5x10^2$) stably expressing cDNA and/or shRNA in complete medium were seeded in each well of a 6-well plate. Plates were incubated at 37 °C for 1 week for A375 and 2 weeks for WM266-4, during which culture medium was refreshed every 3

days for A375 and 1 day for WM266-4, respectively. Following methanol (Merck) fixation and 0.1% crystal violet (Sigma) staining, the number of colonies formed in each well was calculated by Quantity One Software (Bio-Rad).

**AlamarBlue (Proliferation) assay**

Single cell suspension containing $1x10^3$ A375 or WM266-4 melanoma cells was seeded in each well of a 96-well plate and incubated at 37°C. After 24 hours, each well was replaced with 100µL of complete medium containing 10% AlamarBlue (Life Technologies) and incubated at 37°C for 2 hours with light protection. The measurement of the absorbance reading at 570nm and 600nm in each well was recorded for quantification followed by replacement of fresh complete medium. The growth curve of the cells with different treatments was monitored for 4 to 11 days with 6 replicates.

**EDU incorporation staining**

The Click-iT EdU Alexa Fluor 488 Imaging Kit (ThermoFisher) was used. Briefly, before adding the EdU treatment, plated $3\times10^4$ melanoma cells on sterile coverslips in 24-well plate and allowed them to recover overnight. Replace half of the media with fresh media containing 20µM of EdU to the final concentration of 10µM and then incubate 2 hours for A375 and 4 hours for WM266-4 at 37°C, respectively. Afterwards, cells were fixed, permeabilized and incubated with Click-iT reaction cocktail based on the manufacturer’s protocol. The EdU-labeled cells and Hoechst were captured by inverted fluorescent microscope (Nikon). The percentage of positive EdU-stained cells in all Hoechst-labeled cells was presented.

**Transwell invasion assays**

Melanoma cell lines with different treatments were resuspended as single cell ($5x10^4$) in plain medium, then seeded on the transparent PET membrane of cell culture insert (8μm, Falcon) coated with 150uL of Matrigel (2.5mg/mL, Corning). Cells were allowed to invade through the Matrigel and membrane driven by FBS in the lower chamber for 12 hours (A375) or 48 hours (WM266-4). Cells failed to invade were removed by the cotton swap. After 100% methanol fixation and DAPI (1μg/mL, Sigma) staining, the number of positive DAPI signal that representing invaded cells was counted.

**RHOA pull-down activation assay**

The detection of RHOA activity was performed by using RHOA Pull-down Activation Assay Biochem Kits (Cytoskeleton) based on manufacturer's protocol. Melanoma cells at 70% confluence were washed twice with cold PBS and lysed with IP Buffer supplemented with 1% protease and phosphatase inhibitors (ThermoFisher) for 3 minutes on ice. Cell lysates were then centrifuged at 16,000g for 3 min. The supernatant (400μg) was then immediately incubated with Rhotekin-RBD (50μg) beads at 4°C for 2 hours to pull down RHOA-GTP. The beads were then washed three times by wash buffer and subjected to western blotting using mouse monoclonal antibodies against RHOA (Santa Cruz). Total RHOA in whole cell lysates was served as loading control.

**GST pull-down assay**

The fragments of DLC1 (636-1091 aa) and FOXK1 (1-391 aa) were cloned into pGEX-6P- 2 and pET-28a vector, respectively, which were kindly provided by Q Hao from the School of Biomedical Sciences in the University of Hong Kong. GST-tagged DLC1 and His-tagged FOXK1 truncations were transformed into BL21 *E. coli* strain with chloramphenicol resistance.

Competent cells were cultured in 200mL LB medium at 37°C until the OD600 reached 0.6. For protein induction, 0.3mM IPTG was added into the medium and maintained at 16°C for 20 hours. Cell pellets were then resuspended in lysis buffer (20mM Tris-HCl, 150mM NaCl, 1mM DTT) and sonicated to obtain optimal protein concentration, followed by incubation with Glutathione beads (ThermoFisher) at 4°C for 1 hour. GST-bound beads were subsequently incubated with His-tagged FOXK1 fragment for 2 hours and subjected to SDS-PAGE. pGEX-6P-2 empty vector was served as a negative control.

**Fluorescence resonance energy transfer**

$5x10^6$ melanoma cells subjeced to different treatments were trypsinized and washed with PBS, then resuspended in 100µL Resuspension Buffer provided by Neon Transfection System (ThermoFisher), together with 3µg of piggyBac-CAG-FRET-RHOA biosensor plasmid and 1µg of Transposase plasmid. Homogenized single cell suspension was loaded to the Neon pipette plugged on Neon pipette stand. The parameter on Neon device for electroporation was "voltage 1500V, width 10ms, pulses 2". After electroporation, immediately transferred the transfected cells into complete culture medium and seeded on sterile coverslips in 24-well plate. 48 hours post electroporation, cells were fixed by 4% PFA, mounted and proceeded to image acquisition using a Carl Zeiss LSM 780. Images acquisition and processing were based on the methods described previously [16].

**Immunofluorescence**

Following deparaffinization and rehydration, the primary and metastatic melanoma tissue microarray slides were subjected to antigen retrieval in 1X Target Retrieval Solution (Citrate, pH 6.1, Dako) at 100°C for 10 minutes and gradually cooled down

to room temperature for 30 minutes. Sections were blocked by 10% normal goat serum and 1% BSA diluted in 0.1% TritonX-100 and then incubated with primary antibodies at 4°C overnight. For immunostaining on cell lines, $1x10^4$ melanoma cells were seeded onto sterile coverslips in 24-well plate 24 hours prior to 4% formaldehyde fixation on ice for 30 minutes. Cells on coverslip were blocked by 1% BSA with 0.1% TritonX-100 in PBS, followed by 4°C overnight incubation of primary antibodies. Anti-rabbit and anti-mouse antibodies conjugated with Alexa-488, Alexa-Cy3 or Alexa-647 (1:1000, Life Technologies) were applied as secondary antibodies. DAPI (1μg/ml, Sigma) was used as a counter-stain. Fluorescence images were captured using a Carl Zeiss LSM 780 confocal microscope . Line scan and intensity analysis on pixel levels were analyzed by ZEN 2012 (Carl Zeiss) using profile definition feature.

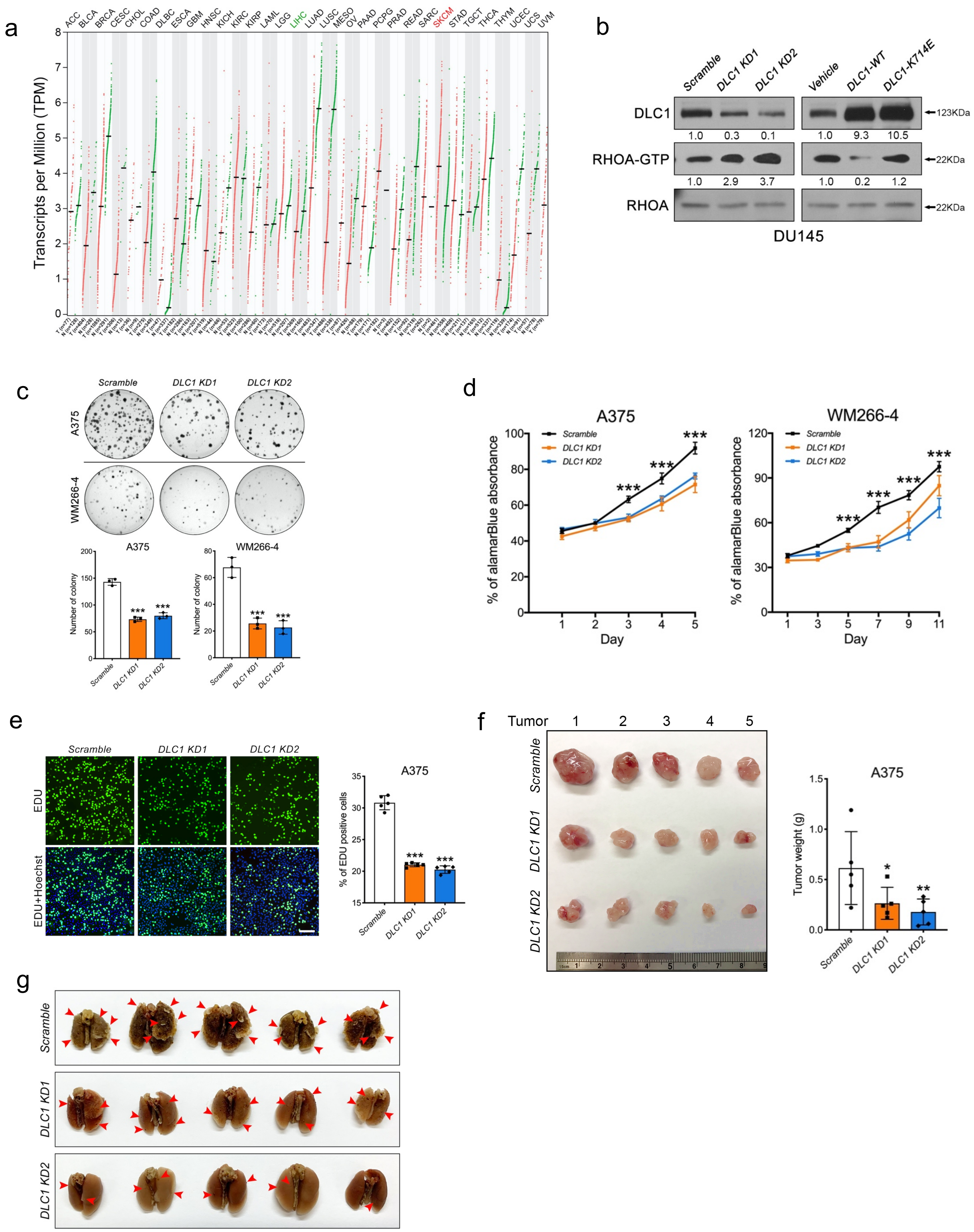

**Supplementary Fig. 1** Increased expression of *DLC1* in skin cutaneous melanomas and DLC1 is required for melanoma growth and metastasis. **(a)** RNA-sequencing data in the TCGA database showing *DLC1* mRNA levels in a variety of cancer samples compared to corresponding normal tissues. **(b)** Immunoblot to detect the level of RHOA-GTP in DU145 prostate cancer cell line subjected to the indicated constructs. *DLC1* KD melanoma cells were subjected to **(c)** clonogenic (n=3) and **(d)** alamarBlue (n=6) assays as well as **(e)** EDU staining to detect proliferating melanoma cells in S phase. Hoechst served as a counterstain. Quantification of EDU positive cells. Scale bar: 100μM. **(f)** Image of xenografts (n=5 per treatment) and their average weight was measured in each treatment group. **(g)** Gross pictures of metastatic lungs from NOD/SCID mice which were injected with the indicated constructs via tail vein. Red arrowheads indicate tumor nodules. * *p*< 0.05, ***p* < 0.01, ****P* < 0.001, by one-way ANOVA. Data represent mean ± SD.

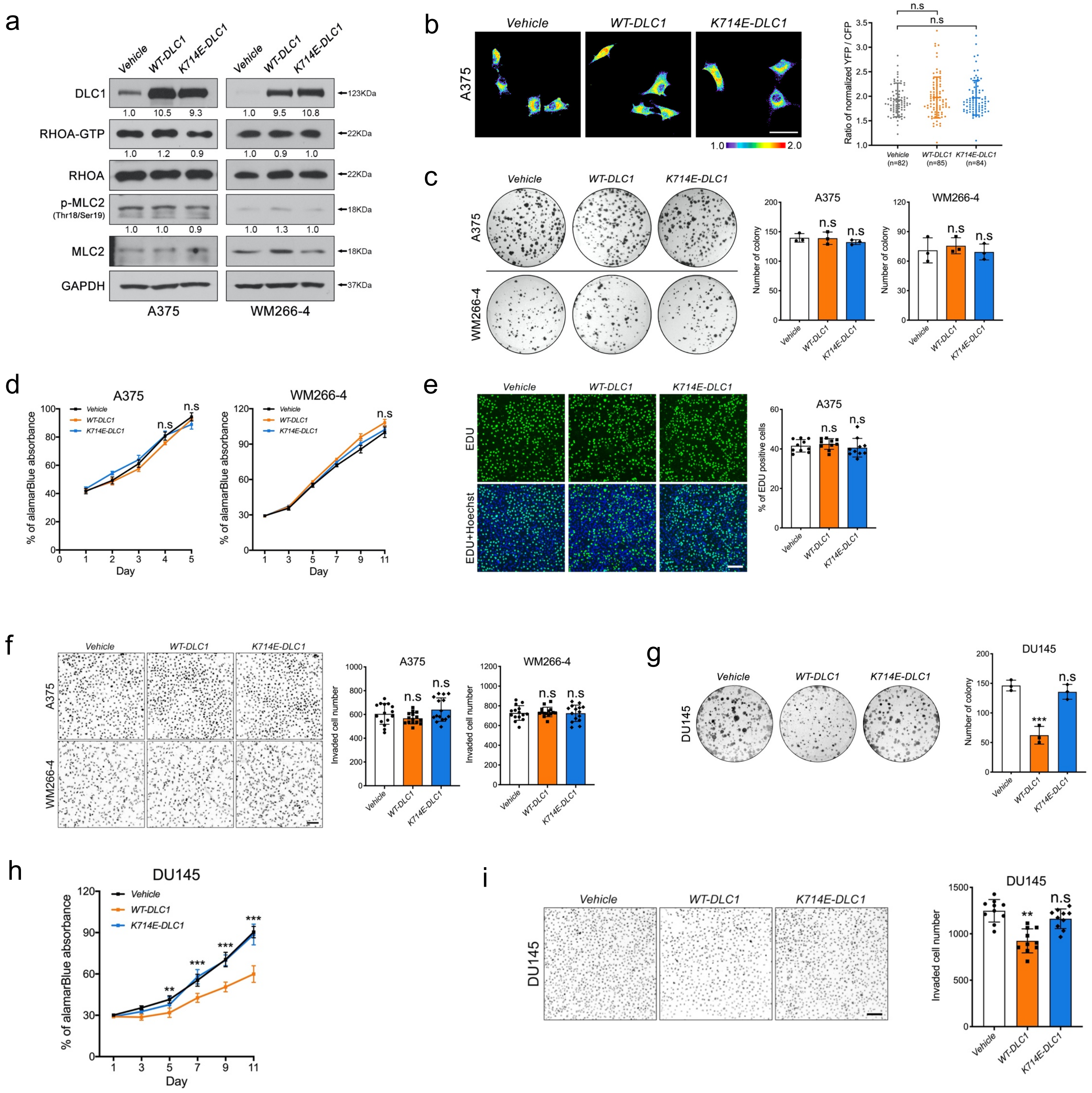


**Supplementary Fig. 2** DLC1 overexpression does not alter RHOA activity and promote melanoma growth, colony formation and metastasis in high DLC1-expressing A375 and WM266-4 melanoma cells. **(a)** Western blot analysis of RHOA-MLC2 pathway in A375 and WM266-4 cells overexpressing with the indicated constructs. GAPDH served as a loading control. **(b)** Representative images of cells expressing RHOA-FRET biosensor. Measurement of RHOA-GTP activity using RHOA-FRET biosensor in cells treated with vehicle control (n=82), WT-DLC1 (n=85) or K714E-DLC1 (n=84). The activity of RHOA-GTP was quantified as a ratio of YFP to CFP. **(c)** Clonogenic (n=3), **(d)** alamarblue (n=6), **(e)** EDU proliferation (n=10) and **(f)** transwell invasion assays (n=15) were conducted in each cell line treated with the indicated constructs. **(g)** Clonogenic (n=3) and **(h)** alamarBlue (n=6) and **(i)** transwell invasion assays (n=10) of DU145 cells transduced with the indicated constructs. Scale bars: 50μM. n.s. non-significant. ** $p < 0.01$, *** $P < 0.001$, by one-way ANOVA analysis. Data represent mean ± SD.

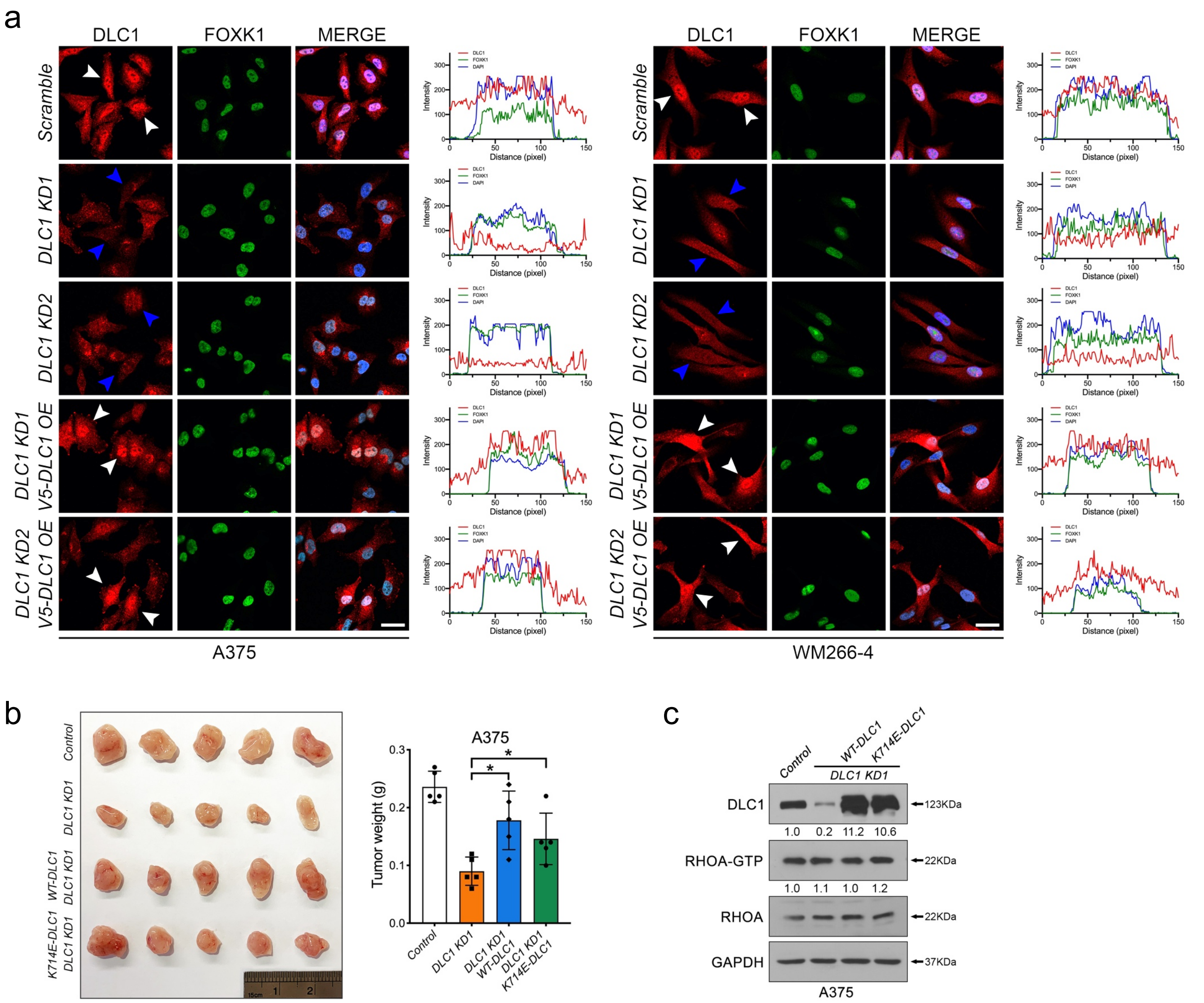


**Supplementary Fig. 3.** Ectopic nuclear DLC1 restores tumor growth in a RHOA independent manner. **(a)** Immunofluorescence showing subcellular localization of endogenous DLC1 in scramble control, *DLC1* KD1 and KD2 cells as well as ectopic DLC1 in *DLC1* KD1 and KD2 cells. White arrowheads indicate nuclear DLC1, whereas blue arrowheads indicate loss of nuclear DLC1 expression in *DLC1* KD1 and KD2 cells. Line scan analysis showing the intensity of nuclear and cytoplasmic DLC1 in cells treated with the indicated constructs. Nuclear FOXK1 was counterstained with DAPI. Scale bars: 50μM. **(b)** Image of xenograft tumors (n=5) and their average weight was measured in each treatment group. **(c)** Immunoblots showing the levels of DLC1 and RHOA-GTP in A375 melanoma cells treated with the indicated constructs. GAPDH served as a loading control. Scale bars: 50μM. p*<0.05. One-way ANOVA was used for statistical analysis. Data represent mean ± SD.

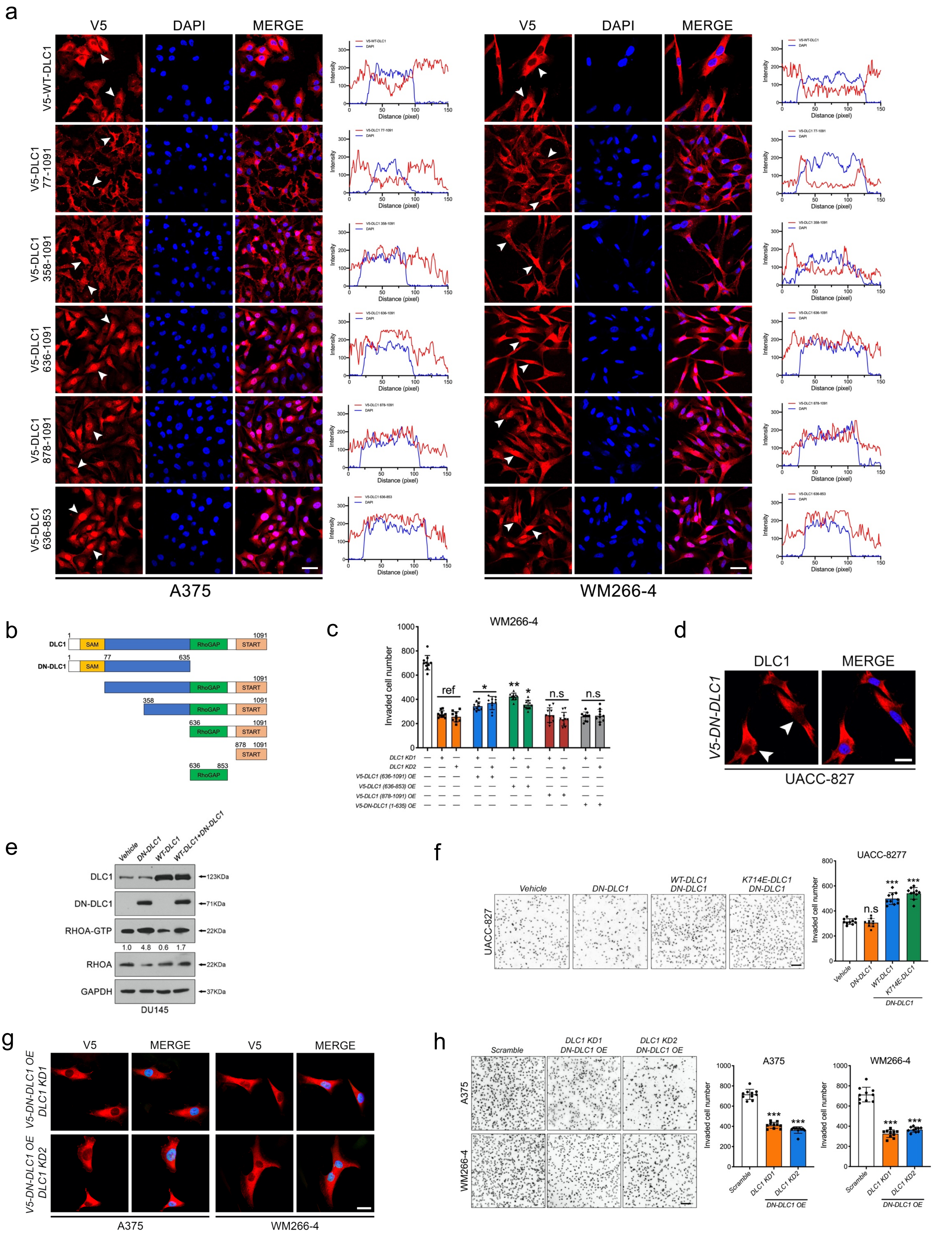


**Supplementary Fig. 4.** Nuclear but not cytoplasmic DLC is oncogenic in melanoma. **(a)** Immunofluorescence showing distinct subcellular localization of V5-DLC1 truncated proteins in melanoma cell lines. Line scan analysis showing the intensity of nuclear and cytoplasmic DLC1 in cells treated with the indicated constructs. **(b)** Schematic of full-length DLC1 protein domains and its truncated constructs. **(c)** Transwell invasion assay showing different degrees of invasiveness of cells treated with the indicated constructs (n=10 per treatment). **(d)** Immunostaining showing the cytoplasmic localization of V5-DN-DLC1 in UACC-827 cells. Nuclei were stained with DAPI. **(e)** Immunoblots showing the levels of RHOA-GTP in DU145 cells treated with the indicated constructs. **(f)** Transwell invasion assay (n=10) of UACC-827 cells transduced with the indicated constructs. **(g)** Immunofluorescence showing ectopic expression of V5-DN-DLC1 in *DLC1 KD1 and KD2* A375 and WM266-4 cells. **(h)** Transwell invasion assays of A375 and WM266-4 cells treated with the indicated constructs. Scale bars: 50μM (e,h) and 100μM (a,g,i). * *p*< 0.05, ***p* < 0.01, ****P* < 0.001, by one-way ANOVA. Data represent mean ± SD.

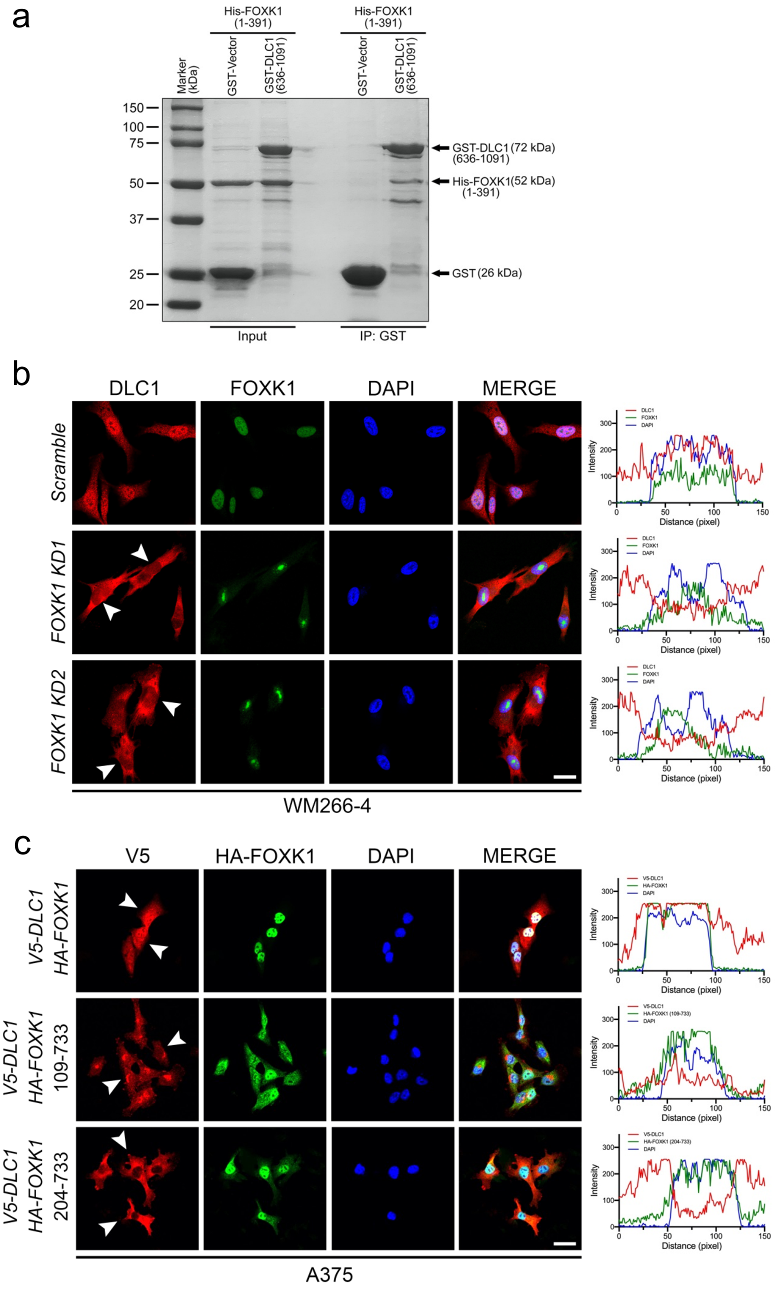


**Supplementary Fig. 5.** FOXK1 promotes DLC1 nuclear translocation and retention through protein-protein interaction. **(a)** GST pull-down showing the direct interaction between GST-tagged DLC1 and His-tagged FOXK1 truncations. GST empty vector served as a negative control. **(b, c)** Immunostaining and intensity line scan analysis showing distinct subcellular localization of DLC1 in cells treated with the indicated constructs. DAPI served as a counterstain. Scale bar: 50μM.

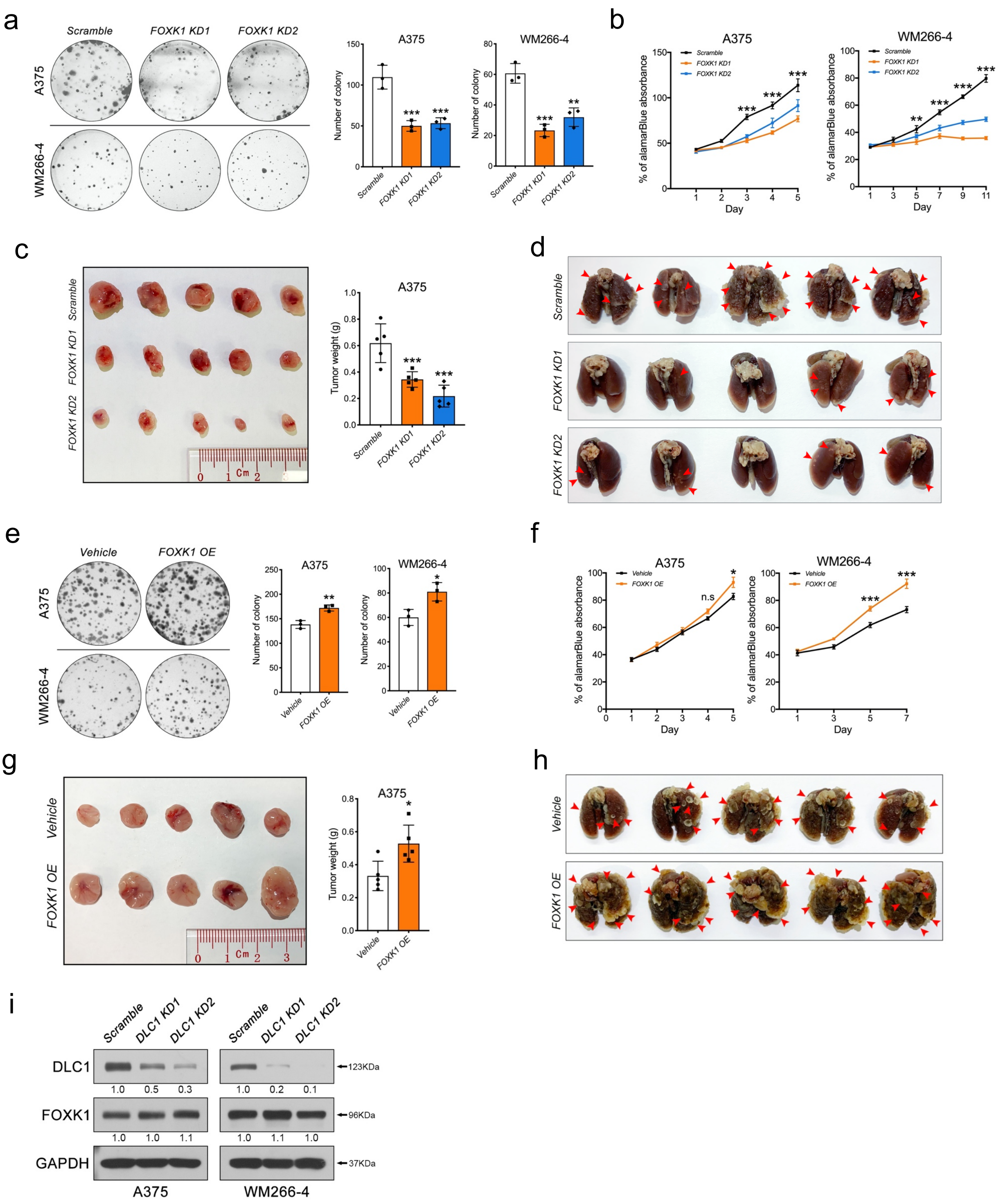


**Supplementary Fig. 6.** FOXK1 promotes melanoma growth and metastasis. **(a, e)** Clonogenic (n=3) and **(b, f)** alamarBlue assays (n=6) of melanoma cells transduced with the indicated constructs. **(c, g)** Images showing subcutaneous xenografts from A375 cells treated with the indicated constructs and quantification of the tumor weight. **(d, h)** Gross images of metastatic lungs from NOD/SCID mice which were injected with the indicated constructs via tail vein (n=5). Red arrowheads indicate tumor nodules. **(i)** Western blots to detect expression of DLC1 and FOXK1 in melanoma cell lines treated with scramble control, *DLC1 KD1* and *DLC1 KD2*. GAPDH served as a loading control. **P* < 0.05, ***P* < 0.01, ****P* < 0.001, by one-way ANOVA. Data represented the mean ± SD.

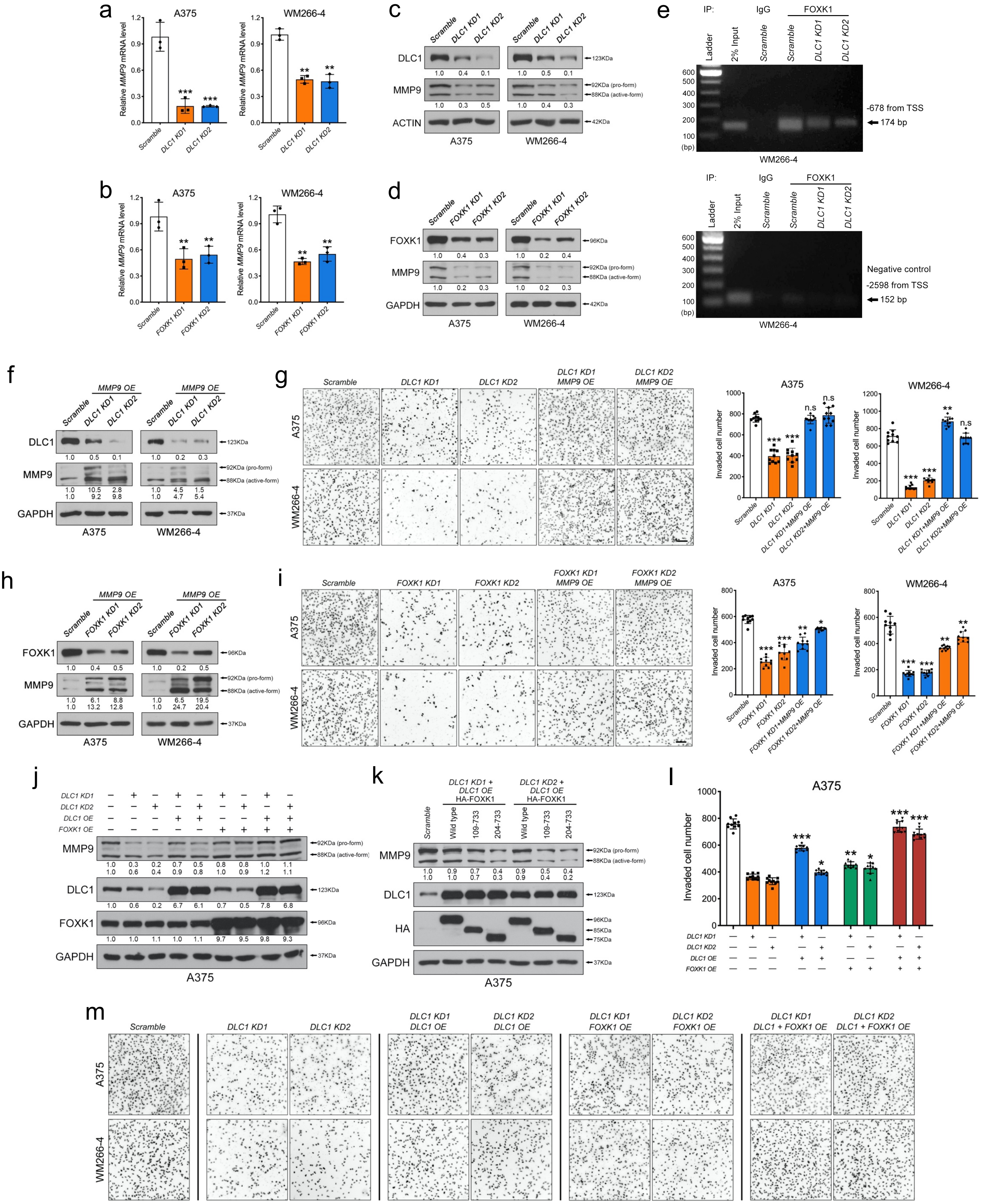

**Supplementary Fig. 7.** DLC1 and FOXK1 cooperatively activate MMP9 expression and promote melanoma invasion and metastasis. **(a, b)** qPCR analysis to confirm the downregulation of *MMP9* expression in *DLC1* KD (n=3) and *FOXK1* KD cells (n=3) compared to scramble control. **(c, d)** Western blot to confirm the reduction of MMP9 expression levels in *DLC1* KD and *FOXK1* KD cells. **(e)** Gel images showing the intensity of ChIP-qPCR products derived from the different treatment groups. IgG served as a negative control. **(f, h)** Immunoblots showing protein levels of *MMP9* OE in *DLC1* KD or *FOXK1 KD* melanoma cells. **(g, i)** Transwell invasion assays (n=10) showing the number of invaded melanoma cells treated with the indicated constructs. **(j, k)** Immunoblots revealing different degrees of restoring MMP9 expression in melanoma cells treated with the indicated constructs. **(l, m)** Transwell invasion assay showing different degree of restoring invasiveness of cells treated with the indicated constructs (n=10 per treatment). Scale bar: 100μM. **P* < 0.05, ***P* < 0.01, ****P* < 0.001, by one-way ANOVA. Data represented the mean ± SD.

# **Supplementary Table 1**. LC-MS analysis of DLC1 pull-down candidates (IgG as control)

| No. | IgG-pull down (1-100) | DLC1-pull down (1-100) | No. | IgG-pull down (101-200) | DLC1-pull down (101-200) | No. | IgG-pull down (201-300) | DLC1-pull down (201-300) |
|---|---|---|---|---|---|---|---|---|
| 1 | HSPA9 | HSPA9 | 101 | CCAR2 | LARP4 | 201 | RBM3 | RPS18 |
| 2 | HNRNPH3 | HNRNPA2B1 | 102 | LARP4 | ZC3HAV1 | 202 | RPLP0 | TRIM25 |
| 3 | HNRNPA2B1 | HNRNPA2B1 | 103 | RPL3 | CT45A3 | 203 | RPL21 | RPS24 |
| 4 | HNRNPA2B1 | HNRPH3 | 104 | RPL10 | ATXN2L | 204 | RPS9 | SYT-SSX1 |
| 5 | HNRNPH1 | HNRNPU | 105 | RPL9 | NONO | 205 | DDX28 | IMPDH2 |
| 6 | HNRNPU | HNRNPH1 | 106 | MRPL11 | RPL9 | 206 | MAP7D1 | STAU2 |
| 7 | HNRNPH3 | HNRNPH3 | 107 | PTBP1 | RPL19 | 207 | CDKN2AIP | ESF1 |
| 8 | MVP | MVP | 108 | SMARCB1 | AGO3 | 208 | MRPS31 | TNRC6B |
| 9 | DDX5 | DDX17 | 109 | PLOD1 | RPL10 | 209 | RPL35 | RPS25 |
| 10 | DDX17 | ILF3 | 110 | RPS4X | MRPL11 | 210 | SFRS3 | RPL35 |
| 11 | DDX17 | HEL-S-72p | 111 | SMARCD2 | JUNB | 211 | STAU2 | TRUB2 |
| 12 | ILF3 | DDX5 | 112 | ZC3HAV1 | ACTL6A | 212 | DHX15 | CEP170 |
| 13 | ILF3 | UBAP2L | 113 | RNMTL1 | HSPD1 | 213 | HP1BP3 | SRSF7 |
| 14 | HEL-S-72p | HNRPA1 | 114 | HIST1H1B | KRT9 | 214 | TUBB6 | RPS7 |
| 15 | YBX1 | YBX1 | 115 | AGO3 | MTDH | 215 | TKT | FXR1 |
| 16 | HNRPA1 | HNRNPL | 116 | SMARCC1 | ATXN2L | 216 | RPL27A | RPL27A |
| 17 | HNRPA1 | HNRNPDL | 117 | CT45A3 | AGO2 | 217 | ATP1A1 | ATAD3B |
| 18 | HNRNPL | HNRPA1 | 118 | LMNA | LARP1 | 218 | MRPS9 | STRAP |
| 19 | HNRNPH2 | HEL-S-89n | 119 | TUBA1C | RPL3 | 219 | RPL28 | DDX24 |
| 20 | HNRNPDL | HNRPK | 120 | UBAP2L | HIST3H2BB | 220 | HEL-S-30 | DDX6 |
| 21 | PABPC1 | PABPC1 | 121 | RPL17 | SMARCD2 | 221 | TNRC6A | RPS21 |
| 22 | HNRPK | YBX1 | 122 | RPSA | hCG_31253 | 222 | RPL12 | SEC23A |
| 23 | HEL-S-89n | HNRNPH2 | 123 | PURA | DDX21 | 223 | RPS5 | BYSL |
| 24 | HNRNPUL2 | HNRNPUL2 | 124 | SEC23B | RPS4X | 224 | RPL18 | SFRS3 |
| 25 | hCG_2020860 | PABPC4 | 125 | SEC23A | RBMS1 | 225 | ERAL1 | MYO1E |
| 26 | HNRNPR | RBM14 | 126 | RPL23A | SMARCB1 | 226 | PCBP2 | HEL-S-30 |
| 27 | PABPC4 | HNRNPA3 | 127 | ZNF326 | KRT10 | 227 | RPL10A | RPS9 |
| 28 | RBM14 | hCG_2020860 | 128 | DDX21 | RPSA | 228 | ADAR | CSDE1 |
| 29 | HNRNPA3 | HNRNPA3 | 129 | FAM168A | FAM168A | 229 | ATAD3B | FXR1 |
| 30 | HNRNPA3 | HNRNPR | 130 | RPS13 | POLR2B | 230 | GPATCH4 | RPS16 |
| 31 | HNRNPD | HNRNPD | 131 | ENO1 | ZNF326 | 231 | EEF2 | CARM1 |
| 32 | IGF2BP1 | HNRNPM | 132 | JUNB | RNMTL1 | 232 | ZC3H7A | PRDX1 |
| 33 | RPS17 | ACTG1 | 133 | RPL5 | SMARCC1 | 233 | RSL1D1 | TPI1 |
| 34 | ELAVL1 | SMARCE1 | 134 | hCG_31253 | PCMT1 | 234 | STRAP | SRSF10 |
| 35 | HSPA9 | DHX9 | 135 | RPL19 | SF3B2 | 235 | DHX36 | DDX47 |
| 36 | CTTN | HNRNPC | 136 | SMARCC2 | RPL23A | 236 | PDIA3 | MRPS26 |
| 37 | SNX9 | RBMX | 137 | RBMS1 | PTBP1 | 237 | TRUB2 | RPS5 |
| 38 | ACTG1 | EWSR1 | 138 | RPL23 | NUFIP2 | 238 | SNRPD1 | TARDBP |
| 39 | HNRPDL | HNRPDL | 139 | PCMT1 | RPL13 | 239 | H2AFY | RPL12 |
| 40 | HNRNPA1 | CTTN | 140 | MTDH | PURA | 240 | HSP90AA1 | ATXN2 |
| 41 | IGF2BP2 | IGF2BP1 | 141 | POLR2B | PRPF19 | 241 | DDX47 | SEC24D |
| 42 | HNRNPM | ELAVL1 | 142 | SF3B2 | SMARCD3 | 242 | SRSF7 | C11orf48 |
| 43 | TUBB4B | HNRNPA1 | 143 | EEF1A1 | RPL17 | 243 | RPL11 | RC3H1 |
| 44 | HNRNPA0 | HEL113 | 144 | HSPD1 | RPL24 | 244 | SRSF10 | MRPS2 |
| 45 | RBMX | CAPRIN1 | 145 | LARP1 | CB4 | 245 | HSPH1 | TAX1BP1 |
| 46 | HEL113 | IGF2BP2 | 146 | RPS19 | RPL29 | 246 | TNRC6B | POLR2C |
| 47 | RTCB | YBX3 | 147 | RPL13 | PLOD1 | 247 | SPATS2 | SEC24B |
| 48 | DPF2 | DAZAP1 | 148 | DDX1 | RPS19 | 248 | SNW1 | DDX28 |
| 49 | YBX3 | HSPA1B | 149 | HIST1H4J | DNAJC10 | 249 | CEP170 | MPG |
| 50 | HNRNPC | RPS17 | 150 | AGO2 | GAPDH | 250 | TPI1 | H2AFY |
| 51 | IGF2BP3 | TFG | 151 | RPL24 | RPL8 | 251 | DDX24 | HEL-S-102 |
| 52 | TUBB | TUBB4B | 152 | RPL8 | SEC23B | 252 | HIST1H2AB | YWHAZ |
| 53 | SYNCRIP | SYNCRIP | 153 | NUFIP2 | SFPQ | 253 | NKRF | MRPS23 |
| 54 | RPS6 | IGF2BP3 | 154 | GAPDH | CCDC15 | 254 | QKI | RPL10A |
| 55 | EWSR1 | HNRNPM | 155 | RPL7A | AGO1 | 255 | NCBP1 | RPS20 |
| 56 | CAPRIN1 | DPF2 | 156 | ZCCHC8 | TKT | 256 | SMARCA4 | TUBB6 |
| 57 | SMARCE1 | KHSRP | 157 | SMARCC2 | HIST1H4J | 257 | RPS21 | ERAL1 |
| 58 | HNRPF | RTCB | 158 | KRT9 | RPS27A | 258 | SEC24D | MRPL15 |
| 59 | KHSRP | DLC1 | 159 | RPL22 | ALDOA | 259 | MRPS7 | HEL-S-271 |
| 60 | DHX9 | TUBB | 160 | UBAP2 | PABPN1 | 260 | RPS25 | PDCD6IP |
| 61 | TFG | YTHDF3 | 161 | RPL29 | RPS13 | 261 | GNL3 | DHX36 |
| 62 | RPS3A | HNRNPA0 | 162 | SF1 | TNRC6A | 262 | MRPL15 | TAGLN2 |
| 63 | HNRNPM | WISP | 163 | RPS7 | RPL5 | 263 | PA2G4 | CHTOP |
| 64 | YTHDF3 | LGALS3BP | 164 | STAU1 | STAU1 | 264 | RPS20 | RPLP0 |
| 65 | HIST1H1D | TAF15 | 165 | PRPF19 | RPS23 | 265 | MYO1E | SRPK2 |
| 66 | HIST1H1C | TUBB4A | 166 | RPS27A | DDX1 | 266 | POTEF | PCBP2 |
| 67 | DAZAP1 | RPS3A | 167 | DNAJC10 | SMARCC2 | 267 | TARDBP | HSPH1 |
| 68 | KRT1 | HIST1H1D | 168 | PUM1 | MAP7D1 | 268 | PDCD6IP | ELAVL2 |
| 69 | UPF1 | HIST1H1C | 169 | SFPQ | ENO1 | 269 | DDX50 | EEF1G |
| 70 | TIA1 | FAM120A | 170 | CARM1 | ATAD3A | 270 | LTV1 | MRPS34 |
| 71 | LGALS3BP | ZFR | 171 | PRDX1 | RPL23 | 271 | RPLP1 | HP1BP3 |
| 72 | RNMT | RPS6 | 172 | HNRNPCL2 | UBAP2 | 272 | SF1 | ZC3H7A |
| 73 | RBMXL1 | TIA1 | 173 | KRT10 | PUM1 | 273 | C11orf48 | P3H1 |
| 74 | NPM1 | NPM1 | 174 | SMARCD3 | DHX15 | 274 | BYSL | SNRPD2 |
| 75 | MATR3 | RBMXL1 | 175 | SRSF9 | SART3 | 275 | RBM14 | RPL18 |
| 76 | FAM120A | EWSR1 | 176 | IMPDH2 | GRN | 276 | SF3A1 | SRP14 |
| 77 | RPS3 | SNRPA | 177 | PCBP1 | RPL14 | 277 | TUFM | PLAT |
| 78 | RPS2 | RALY | 178 | RPS24 | HIST1H3A | 278 | HEL-S-271 | RPL28 |
| 79 | NCL | NCL | 179 | DHX30 | ZCCHC8 | 279 | ANXA2 | RPS12 |
| 80 | TAF15 | G3BP1 | 180 | ATXN2L | KRT2 | 280 | FOXC2 | FXR2 |
| 81 | RPS8 | MATR3 | 181 | MOV10 | RSL1D1 | 281 | ESF1 | UBE1 |
| 82 | EWSR1 | UPF1 | 182 | CB4 | ADAR | 282 | MRPS28 | MRPS7 |
| 83 | ZFR | RPS11 | 183 | RPS18 | RPLP1 | 283 | CHTOP | SF1 |
| 84 | SEC24C | RPS8 | 184 | HIST1H3A | SF1 | 284 | TRNAU1AP | DAP3 |
| 85 | TUBB3 | SEC24C | 185 | HSP90AB1 | RBMS2 | 285 | RPL32 | FAM103A1 |
| 86 | RPS11 | RNMT | 186 | ATXN2L | HNRNPCL1 | 286 | RPS15 | CTNND1 |
| 87 | SMARCD1 | SMARCD1 | 187 | ATAD3A | HSP90AB1 | 287 | KPNA1 | CTAG1B |
| 88 | RPL26 | HNRPF | 188 | RPS23 | RPL7A | 288 | GRL1 | RBM14 |
| 89 | NONO | CCAR2 | 189 | MRPS22 | DHX30 | 289 | FXR2 | PYM1 |
| 90 | SNRPA | KRT1 | 190 | GRN | HSP90AA1 | 290 | RPS12 | PSMA1 |
| 91 | G3BP1 | RPS2 | 191 | TRIM25 | RBM10 | 291 | PGK1 | YWHAQ |
| 92 | HIST1H2BN | HIST1H2BN | 192 | RBMS2 | RPL11 | 292 | POLR2C | C7orf11 |
| 93 | AKAP8 | ALYREF | 193 | SART3 | PDIA3 | 293 | SYT-SSX1 | SPATS2 |
| 94 | TUBA1B | AKAP8 | 194 | DDX6 | PCBP1 | 294 | HIST2H3PS2 | USP10 |
| 95 | RALY | LMNA | 195 | RPL14 | RPL7 | 295 | MRPS23 | QKI |
| 96 | RPL26L1 | RPL26 | 196 | RPS16 | RBM3 | 296 | DNAJC9 | ARID1A |
| 97 | ALYREF | TUBA1A | 197 | PABPN1 | MOV10 | 297 | RPUSD3 | MRPS9 |
| 98 | KHDRBS1 | RPS3 | 198 | ALDOA | MRPS22 | 298 | BCKDK | DLX2 |
| 99 | ACTL6A | RPS14 | 199 | RPL7 | RPL22 | 299 | KRT2 | RPS15 |
| 100 | RPS14 | HIST1H1B | 200 | HSP90B1 | CDKN2AIP | 300 | MRPS2 | ATXN2 |

**Supplementary Table 1**. (Continued)

| No. | IgG-pull down (301-400) | DLC1-pull down (301-400) | No. | IgG-pull down (401-500) | DLC1-pull down (401-500) | No. | IgG-pull down (501-600) | DLC1-pull down (501-600) |
|---|---|---|---|---|---|---|---|---|
| 301 | NGRN | HIST2H3PS2 | 401 | LYAR | NOP2 | 501 | CHCHD3 | CSTB |
| 302 | SQSTM1-ALK | ATP1A1 | 402 | G3BP2 | GNL3L | 502 | DDX27 | RBBP7 |
| 303 | MRPS26 | ANXA1 | 403 | RPL13A | RPL27 | 503 | MDH2 | YWHAE |
| 304 | CLTA | EEF2 | 404 | RPS28 | RPS27L | 504 | PDCD6 | MRPL19 |
| 305 | DAP3 | DDX27 | 405 | PRPF39 | UTP14A | 505 | S1 | MRPS11 |
| 306 | PLAT | HEL-S-123m | 406 | YWHAG | ABI2 | 506 | SAV1 | RPS18 |
| 307 | ZCCHC9 | NCOA5 | 407 | LOXL2 | GPATCH4 | 507 | MYEF2 | ZCCHC9 |
| 308 | ELAVL2 | NGRN | 408 | FAU | RPS28 | 508 | HEL-S-69 | PDIA6 |
| 309 | RBBP4 | CKAP4 | 409 | KRT5 | SRSF1 | 509 | P4HB | DUT |
| 310 | PHGDH | IFI16 | 410 | XRCC6 | KIF1B | 510 | SMAD9 | NGDN |
| 311 | CTAG1B | WBSCR16 | 411 | AKAP8L | AKAP8L | 511 | SNRPB | PTRF |
| 312 | FXR1 | CCDC86 | 412 | hCG_2042749 | DDX52 | 512 | RFC4 | RUVBL1 |
| 313 | RBM8 | LSM14B | 413 | RPS27L | MRPL44 | 513 | PRMT6 | MRPL40 |
| 314 | ZNF346 | RPUSD3 | 414 | ANXA1 | HEL-S-69 | 514 | GTPBP4 | MRPL49 |
| 315 | SPATS2L | ANXA2 | 415 | ZFP36L1 | PUM2 | 515 | MEX3C | SAV1 |
| 316 | SDHA | GADD45GIP1 | 416 | LDHB | MKRN1 | 516 | EIF4H | PHAX |
| 317 | RBM10 | FOXC2 | 417 | ZC3H11A | MRPS35 | 517 | GNL2 | CDC40 |
| 318 | USP10 | LTV1 | 418 | YWHAB | RPS27 | 518 | SMARCA5 | HIST2H2BC |
| 319 | BCL7C | DDX50 | 419 | KPNA6 | RPL38 | 519 | SFRS4 | PURB |
| 320 | DDB1 | EBNA1BP2 | 420 | H1F0 | SNRPA1 | 520 | NIFK | EIF6 |
| 321 | SNRPD2 | TOP1 | 421 | RPL34 | LPP | 521 | EXOSC6 | IGBP1 |
| 322 | HEL-S-123m | ZCCHC3 | 422 | BTF3 | SMNDC1 | 522 | CDKN2AIPNL | SMARCA4 |
| 323 | H1FX | GNL3 | 423 | RNPS1 | DDB1 | 523 | SNRPD3 | HAX1 |
| 324 | ZNF800 | SPICE1 | 424 | RPS18 | NUP88 | 524 | PPM1F | MDH2 |
| 325 | PDIA3 | EIF4A2 | 425 | CCDC86 | SOX10 | 525 | scFv | BOP1 |
| 326 | SRP14 | RUNX1 | 426 | LPP | PHF8 | 526 | N-PAC | SERBP1 |
| 327 | PLEC | PDIA3 | 427 | SRRT | RBM15 | 527 | MARK3 | PGAM5 |
| 328 | MARK2 | MRPS31 | 428 | NOP16 | NUP98 | 528 | SLC25A13 | DDX55 |
| 329 | SEC24B | PLRG1 | 429 | HEL-S-97n | SMARCA5 | 529 | PFN2 | JUND |
| 330 | RPLP1 | SMARCA2 | 430 | POP1 | RCC2 | 530 | RFC1 | DSG1 |
| 331 | FAM103A1 | HEL-S-22 | 431 | CBX3 | PHF6 | 531 | SCYL1 | SFRS5 |
| 332 | KIFC1 | NKRF | 432 | RBM15 | MRPS25 | 532 | DKFZp666D193 | CDC73 |
| 333 | MRPS11 | CD3EAP | 433 | POLR2A | LRRC47 | 533 | SOX10 | ZYX |
| 334 | LSM12 | EXOSC6 | 434 | SRSF1 | BCL7C | 534 | SPFH1 | CSE1L |
| 335 | IFI16 | ACTN4 | 435 | NOP2 | RPL36 | 535 | IGHV1-45 | TCEB1 |
| 336 | CHERP | YWHAB | 436 | FOSL2 | DCTPP1 | 536 | ZNF385A | SS18L1 |
| 337 | CNN3 | SF3A1 | 437 | MTERF3 | SRRT | 537 | SLC25A3 | PPM1F |
| 338 | FAM98A | CIRBP | 438 | PDE12 | IGHMBP2 | 538 | SERBP1 | PAIP1 |
| 339 | RPL38 | GRL1 | 439 | CKAP4 | POLR2I | 539 | NAT10 | SH3PXD2A |
| 340 | NUP98 | YTHDF2 | 440 | XRCC5 | TCF20 | 540 | PCNA | PRPF39 |
| 341 | FTSJ3 | PSIP1 | 441 | TOE1 | SLC25A5 | 541 | DDX54 | PFN2 |
| 342 | PURB | KPNA1 | 442 | U2SURP | TRA2A | 542 | HEL-S-7 | MEX3C |
| 343 | ARID1A | CDC37 | 443 | GIPC1 | DKC1 | 543 | HEL-S-70 | SRPRB |
| 344 | PSIP1 | YWHAG | 444 | SF3B1 | XRCC5 | 544 | HOXC4 | TIMP3 |
| 345 | IGHMBP2 | GTF3C4 | 445 | EIF2S1 | KRR1 | 545 | RPUSD4 | CANX |
| 346 | HEL-S-102 | HEL-S-2a | 446 | MRPL39 | DKFZp666D193 | 546 | SF3B5 | MPHOSPH10 |
| 347 | PUM2 | CLTA | 447 | KRT16 | TNRC6C | 547 | RPL37A | DRG1 |
| 348 | PYM1 | POP1 | 448 | TOP1 | EEF1D | 548 | MPHOSPH10 | G6PD |
| 349 | RPL36 | SNW1 | 449 | UQCRC2 | AATF | 549 | DCTPP1 | SSB |
| 350 | RPS15A | MRPL13 | 450 | H2AFZ | NUMB | 550 | HIST2H2BC | FIP1L1 |
| 351 | HEL-S-2a | FAM120C | 451 | MRPL14 | FTSJ3 | 551 | MRPL17 | MDH1 |
| 352 | PLRG1 | TSR1 | 452 | ASPH | BCL7B | 552 | CDC37 | NCBP2 |
| 353 | YTHDF2 | RSBN1L | 453 | MRPS5 | LDHB | 553 | PRDX5 | NUFIP1 |
| 354 | MRPS34 | SEC24A | 454 | MRPL43 | TUFM | 554 | MRPL49 | SLC25A22 |
| 355 | GADD45GIP1 | EMD | 455 | ACTN1 | MRPS5 | 555 | CCT2 | RAE1 |
| 356 | SEC16A | CKAP2L | 456 | RPS27 | FBL | 556 | PTCD3 | PIPSL |
| 357 | WBSCR16 | RPL36A | 457 | SNRPA1 | NIFK | 557 | SP2 | PDE12 |
| 358 | KRT6B | hCG_2042749 | 458 | TRIP6 | LDHA | 558 | PDIA6 | PHF5A |
| 359 | ARHGEF2 | SNR | 459 | ZNF574 | PGK1 | 559 | MRPS6 | GATAD2A |
| 360 | RPL30 | EIF4H | 460 | C7orf11 | ABT1 | 560 | DKFZp686E1899 | KYNU |
| 361 | SCAMP3 | SCAMP3 | 461 | MRPS25 | MRPS16 | 561 | NHP2 | RPUSD4 |
| 362 | PTRF | G3BP2 | 462 | SLC25A5 | RBBP4 | 562 | DLX1 | PHGDH |
| 363 | THRAP3 | SF3B1 | 463 | DPF1 | LOXL4 | 563 | SECISBP2 | WDR74 |
| 364 | EIF4A2 | SEC16A | 464 | LARP4B | ARHGEF2 | 564 | MRPS33 | TOX4 |
| 365 | ATXN2 | BTF3 | 465 | FIP1L1 | U2SURP | 565 | ZGPAT | P4HB |
| 366 | UBA52 | MRPL4 | 466 | YWHAQ | NCBP3 | 566 | DUSP11 | H1FX |
| 367 | RBM7 | FAU | 467 | CCT8 | GCOM1 | 567 | SSBP1 | PUF60 |
| 368 | KRT14 | FAM98A | 468 | MRPS14 | TCEB3 | 568 | CFL1 | RAN |
| 369 | TSR1 | RPL32 | 469 | MRPS16 | SPFH1 | 569 | SUPT5H | ISY1 |
| 370 | EEF1D | WDR6 | 470 | BCAS2 | SART1 | 570 | PSMA1 | PLEC |
| 371 | TAX1BP1 | RBM7 | 471 | COPG1 | COPG1 | 571 | RRS1 | POP7 |
| 372 | LSM14B | TRNAU1AP | 472 | ABT1 | UQCRC2 | 572 | EXOSC10 | SUPT16H |
| 373 | MRPL13 | POLR2A | 473 | RPS26 | CFL1 | 573 | RACK1 | RNH1 |
| 374 | GTF3C4 | MRPS28 | 474 | NCBP3 | MARK2 | 574 | NELFE | FMNL3 |
| 375 | DDX10 | DDX10 | 475 | ISY1 | ZNF346 | 575 | TIMP3 | SF3B5 |
| 376 | NCBP2 | TPX2 | 476 | KNOP1 | CTCF | 576 | AK2 | MAGEA10 |
| 377 | CSDE1 | LOXL2 | 477 | MRPS35 | KRT16 | 577 | PIPSL | SNU13 |
| 378 | MRPL1 | HSP90B1 | 478 | CTPS1 | ZFP36L1 | 578 | UTP14A | C19orf53 |
| 379 | SRPK1 | CNN3 | 479 | ACLY variant protein | BCL7A | 579 | MPG | CRBN |
| 380 | NCOA5 | KPNA6 | 480 | TSPYL | SRPK1 | 580 | KHDRBS3 | PHC2 |
| 381 | SEC24A | MRPL39 | 481 | DKC1 | HOXC4 | 581 | PHF5A | NAP1L1 |
| 382 | YWHAZ | XRCC6 | 482 | HMGA1 | ABCF1 | 582 | ZNF281 | RACGAP1 |
| 383 | RCC2 | SNRPD1 | 483 | MRPL23 | ZC3H11A | 583 | LSM4 | TRIM56 |
| 384 | KRR1 | CHERP | 484 | HMG20A | CDKN2AIPNL | 584 | RBM47 | PDLIM4 |
| 385 | RPL27 | SSBP1 | 485 | PHF6 | GSN | 585 | EIF6 | MRPL1 |
| 386 | SMARCA2 | PFN1 | 486 | RSBN1L | HMG20A | 586 | G6PD | LSM12 |
| 387 | SNR | SFRS4 | 487 | LOXL4 | DDX18 | 587 | CD3EAP | C8orf33 |
| 388 | MRPL4 | RBM39 | 488 | REPIN1 | RNPS1 | 588 | hCG_1984214 | SCYL1 |
| 389 | LRRC47 | PA2G4 | 489 | MTA2 | RPS26 | 589 | TINAGL1 | PRKCDBP |
| 390 | RBM39 | ZNF385A | 490 | DUT | DDX54 | 590 | GCOM1 | MRPL17 |
| 391 | CIRBP | NCBP1 | 491 | KIF1B | NAT10 | 591 | C15orf52 | ACLY variant protein |
| 392 | TPX2 | SUPT5H | 492 | LDHA | EIF2S1 | 592 | SLC1A5 | ZNF207 |
| 393 | ZCCHC3 | RBM4B | 493 | MCCC2 | MTA2 | 593 | NOP58 | CBX3 |
| 394 | EBNA1BP2 | KIFC1 | 494 | TAGLN2 | SNRPD3 | 594 | RBM17 | ZNF697 |
| 395 | MRPL44 | SPATS2L | 495 | MRPL47 | MBNL1 | 595 | CKAP2L | LSM14A |
| 396 | EMD | SQSTM1-ALK | 496 | HSPA4 | CHCHD3 | 596 | FBL | CLIC1 |
| 397 | DLX2 | ZNF800 | 497 | RAE1 | H1F0 | 597 | YWHAE | RBM8 |
| 398 | PHB | RPL13A | 498 | NAP1L1 | RIOK2 | 598 | RTCA | EIF2AK2 |
| 399 | RPL36A | TRIP6 | 499 | TRA2A | RNF2 | 599 | RAB7A | COLGALT1 |
| 400 | RBM4B | KRT5 | 500 | RBM42 | NOP16 | 600 | MAT2A | AIP |

## **Supplementary Table 1**. (Continued)

| No. | IgG-pull down (601-700) | DLC1-pull down (601-700) | No. | IgG-pull down (701-768) | DLC1-pull down (701-791) |
|---|---|---|---|---|---|
| 601 | CTNND1 | NOP58 | 701 | DNAJB11 | LMNB1 |
| 602 | METAP2 | PPIL2 | 702 | SRPRB | RRP9 |
| 603 | SS18L1 | SPTY2D1 | 703 | TCF12 | HEL-S-128m |
| 604 | LSM14A | SMTN | 704 | TRIM56 | LARP7 |
| 605 | RBM45 | H2AFZ | 705 | TARS2 | ABI1 |
| 606 | WDR74 | SP1 | 706 | NUMB | UTP3 |
| 607 | PHF2 | NHP2 | 707 | HEL-S-128m | GNL2 |
| 608 | DRG1 | THRAP3 | 708 | ITCH | MRPS27 |
| 609 | FAM120C | ARHGDIA | 709 | TFIP11 | SRI |
| 610 | SFRP1 | ZC3H4 | 710 | EZR-ROS1 | RRP15 |
| 611 | SEMA3B | TFIP11 | 711 | CTNNB1 | MIF |
| 612 | EHD1 | JTV1 | 712 | ARHGDIA | SHMT2 |
| 613 | CRBN | MRPL23 | 713 | CTCF | HMGA1 |
| 614 | MAGEA10 | TSPYL | 714 | TRMT2A | RBM28 |
| 615 | MGC3731 | EIF3D | 715 | MAGOH | RBM42 |
| 616 | UBE1 | SNRPB | 716 | PQBP1 | ETV3 |
| 617 | p65 | DNAJC21 | 717 | VAT1 | HEL-S-45 |
| 618 | SSB | RSBN1 | 718 | MRPL40 | ZGPAT |
| 619 | HADHB | FOXK1 | 719 | MKRN1 | EXOSC8 |
| 620 | POP7 | TOE1 | 720 | RC3H1 | SMU1 |
| 621 | SMTN | BOLA2 | 721 | MMTAG2 | MEX3A |
| 622 | FRG1 | p65 | 722 | C19orf53 | EFHD2 |
| 623 | CSTB | NOL9 | 723 | LMNB1 | CRNKL1 |
| 624 | TNRC6C | TROVE2 | 724 | MRPL2 | SMAD9 |
| 625 | PFN1 | CTNNA1 | 725 | CALU | ARHGEF1 |
| 626 | PKP3 | CSTF1 | 726 | TMA16 | HEL-S-70 |
| 627 | LASP1 | RBM34 | 727 | HEL-S-80p | RBFOX2 |
| 628 | CRIP2 | EIF5A | 728 | MIF | DBN1 |
| 629 | IGBP1 | GPN1 | 729 | RPRD1B | MYL12A |
| 630 | ATP5C1 | MRPS21 | 730 | TXN | KRT12 |
| 631 | PSPC1 | PHLDA2 | 731 | ZNF593 | NDE1 |
| 632 | MGME1 | CCT2 | 732 | DNAJA1 | TCEB2 |
| 633 | CDC73 | LASP1 | 733 | DCAF13 | RFC2 |
| 634 | CLINT1 | CSRP1 | 734 | BOP1 | SLC25A3 |
| 635 | CSTF1 | PRDX5 | 735 | BOLA2 | DNAJC9 |
| 636 | GPATCH2 | PRPF38A | 736 | NUDC | CTNNB1 |
| 637 | SRPK2 | FSCN1 | 737 | HDGF | PPP1R14B |
| 638 | PRKCDBP | CCDC84 | 738 | ZC3H7B | Nbla00360 |
| 639 | CSRP1 | CBX5 | 739 | MRPL19 | MRPL21 |
| 640 | STOML2 | METAP2 | 740 | IVNS1ABP | MRPL18 |
| 641 | NGDN | PARK7 | 741 | LENG8 | BCAS2 |
| 642 | CIRBP | PTBP3 | 742 | ZNHIT3 | RBM47 |
| 643 | PDHB | SDAD1 | 743 | EIF2S3 | MRPS33 |
| 644 | GPN1 | CPSF7 | 744 | SF3A2 | SLC4A1AP |
| 645 | IMMT | CTPS2 | 745 | KIAA0971 | DCAF13 |
| 646 | NME1 | RFC1 | 746 | URI1 | SF3A2 |
| 647 | RPS29 | MYEF2 | 747 | BCL7A | RCHY1 |
| 648 | XPC | DNAJA1 | 748 | DKFZp686M24218 | CWF19L1 |
| 649 | EIF3D | RPL34 | 749 | U2AF2 | DUSP11 |
| 650 | JTV1 | CASC3 | 750 | AIMP1 | FRMD4A |
| 651 | SPTY2D1 | LSM4 | 751 | TDRD3 | LRPPRC |
| 652 | CLTC | RPL15 | 752 | MRPL21 | AHR |
| 653 | MAML1 | NUDT21 | 753 | SEC22B | HMGB1 |
| 654 | AHCY | ZNF593 | 754 | PPIB | SLC1A5 |
| 655 | ZCRB1 | MYL6 | 755 | RCN2 | CD70 |
| 656 | RAN | BMP2K | 756 | MUS81 | CACYBP |
| 657 | RPL15 | SH3BGRL3 | 757 | FAM83D | NOLC1 |
| 658 | PRPF31 | TCP1 | 758 | PFDN2 | PTCD3 |
| 659 | AIP | EXOSC10 | 759 | TMEM113 | VAT1 |
| 660 | PYCR2 | INCENP | 760 | AHR | KHDRBS3 |
| 661 | P3H1 | TALDO1 | 761 | UTP3 | SEC31A |
| 662 | CBX8 | MARK3 | 762 | UPF3B | UPF3B |
| 663 | CRNKL1 | IMMT | 763 | NUP88 | CMSS1 |
| 664 | LARP7 | CTPS1 | 764 | PHC2 | LENG8 |
| 665 | RPL39 | ACAN | 765 | SRPX | RBM45 |
| 666 | PPAN-P2RY11 | NFIB | 766 | EXOSC9 | ADARB1 |
| 667 | KLHL12 | MTERF3 | 767 | PARK7 | SRPX |
| 668 | LARP1B | RANBP1 | 768 | CMSS1 | EIF2S3 |
| 669 | PRPF6 | PARN | 769 |  | RPL30 |
| 670 | PAICS | SRP9 | 770 |  | CYR61 |
| 671 | PDLIM4 | SLC25A13 | 771 |  | KIF5B-ALK |
| 672 | TROVE2 | DPF1 | 772 |  | LGALS1 |
| 673 | MAPK1 | MAP3K7 | 773 |  | HEL-S-99n |
| 674 | EIF2AK2 | RPL39 | 774 |  | TRPT1 |
| 675 | ATP2A2 | UBE2NL | 775 |  | PRCC |
| 676 | PLS3 | PPAN-P2RY11 | 776 |  | PHB |
| 677 | SMNDC1 | N-PAC | 777 |  | RPL35A |
| 678 | SEC13 | HADHB | 778 |  | SH3GL1 |
| 679 | PAIP1 | KIAA0971 | 779 |  | ZC3H7B |
| 680 | MRPL46 | RPS29 | 780 |  | HS1-MOB4 |
| 681 | JUND | RARS | 781 |  | hCG_17415 |
| 682 | GATAD2A | SUGP2 | 782 |  | RPRD1B |
| 683 | RBM28 | GPATCH11 | 783 |  | RBM15B |
| 684 | RPL35A | LARP1B | 784 |  | GFPT1 |
| 685 | NUDT21 | POLR2G | 785 |  | RPIA |
| 686 | EXOSC4 | CYFIP2 | 786 |  | MRPL53 |
| 687 | RPN1 | TOR1AIP1 | 787 |  | C14orf166 |
| 688 | PRKRA | DDX41 | 788 |  | AIMP1 |
| 689 | PGAM5 | PQBP1 | 789 |  | ATXN2 |
| 690 | NOL10 | CCT8 | 790 |  | MRPL46 |
| 691 | ACAN | TARS2 | 791 |  | ZNHIT3 |
| 692 | GSN | C15orf52 |  |  |  |
| 693 | MAP3K7 | CD2BP2 |  |  |  |
| 694 | SRFBP1 | DNAJB6 |  |  |  |
| 695 | FKBP9 | HEL-S-7 |  |  |  |
| 696 | PHAX | SEC13 |  |  |  |
| 697 | LUZP1 | RBMS3 |  |  |  |
| 698 | NUDT1 | FKBP9 |  |  |  |
| 699 | USP15 | LARP4B |  |  |  |
| 700 | EEF1G | PRPF6 |  |  |  |

**Supplementary Table 2** List of shRNA oligos and qPCR primers

| **Name** | **Sequence (5' – 3')** |
|---|---|
| *shDLC1* KD1 | CCTTGACTGGAATATGTAA |
| *shDLC1* KD2 | CCCGATTGCAAATAGTGAT |
| *shFOXK1* KD1 | CCATCAAGATCCAGTTCACGT |
| *shFOXK1* KD2 | GCTGCTATGAAGACAGGATTA |
| *shMMP9* KD2 | CCACAACATCACCTATTGGAT |
| qPCR-*DLC1*-For | CACAGGACAACCGTTGCCTCGA |
| qPCR-*DLC1*-Rev | CTCTTCAGGGTGTTGAGATGGA |
| qPCR-*FOXK1*-For | ACACGTCTGGAGGAGACAGC |
| qPCR-*FOXK1*-Rev | GAGAGGTTGTGCCGGATAGA |
| qPCR-*MMP9*-For | ACGATGACGAGTTGTGGTCC |
| qPCR-*MMP9*-Rev | TGTAGAGTCTCTCGCTGGGG |
| qPCR-*36B4*-For | GTGATGTGCAGCTGATCAAGACT |
| qPCR-*36B4*-Rev | GAAGACCAGCCCAAAGGAGA |
| ChIP-Negative-For | GACAGCCCCAAGTGCCAATA |
| ChIP-Negative-Rev | CCCCCACTTGCCATCAATG |
| ChIP-FOXK1 motif-For | CATTTGCCCGAGGTCCTGAA |
| ChIP-FOXK1 motif-Rev | GTCTTCCGCAGGCTGAATCT |

**Supplementary Table 3** List of primary antibodies used in this study

| Name of antibody | Manufacturer | Species | Dilution (IB) | Dilution (IF) |
|---|---|---|---|---|
| DLC1 | 612020, BD Biosciences | Mouse | 1:500 | — |
| DLC1 (C-12) | sc-271915, Santa Cruz | Mouse | — | 1:100 |
| FOXK1 (ChIP-grade) | ab18196, Abcam | Rabbit | 1:5000 | 1:1000 |
| FOXK1 (G-4) X | sc-373810X, Santa Cruz | Mouse | 1:5000 | 1:1000 |
| MMP9 | ab38898, Abcam | Rabbit | 1:1000 | 1:500 |
| SOX10 | sc-17342, Santa Cruz | Goat | 1:500 | 1:200 |
| p-MLC2 (Thr18/Ser19) | ab3674, Cell Signaling | Rabbit | 1:500 | — |
| MLC2 | sc-517244, Santa Cruz | Mouse | 1:500 | — |
| RHOA | sc-418, Santa Cruz | Mouse | 1:500 | — |
| V5 | R960-25, ThermoFisher | Mouse | 1:5000 | 1:1000 |
| HA | ab9110, Abcam | Rabbit | 1:5000 | 1:1000 |
| GAPDH | sc-25778, Santa Cruz | Rabbit | 1:5000 | — |
| ß-ACTIN | A2228, Sigma Aldrich | Mouse | 1:5000 | — |
